# Metabolic pathway analysis reveals hierarchical pentose sugar utilization and metabolic flexibility of *Bifidobacterium longum*

**DOI:** 10.1080/19490976.2026.2647591

**Published:** 2026-03-23

**Authors:** Lisa Friess, Fionnuala M. McAuliffe, Paul D. Cotter, Anthony L. Shiver, Kerwyn Casey Huang, Anne de Jong, Douwe van Sinderen

**Affiliations:** a APC Microbiome Ireland & School of Microbiology, University College Cork, Cork, Ireland; b UCD Perinatal Research Centre, School of Medicine, University College Dublin, National Maternity Hospital, Dublin, Ireland; c Teagasc Food Research Centre, Cork, Ireland; d Department of Bioengineering, Stanford University, Stanford, USA; e Department of Microbiology and Immunology, Stanford University School of Medicine, Stanford, USA; f Chan-Zuckerberg Biohub, San Francisco, USA; g Department of Molecular Genetics, University of Groningen, Groningen, the Netherlands

**Keywords:** Arabinose, xylose, ribose, probiotic, prebiotic, bifid shunt, bifidobacteria

## Abstract

Plant-derived pentose sugars represent a major nutrient source in the gut, yet their metabolism remains incompletely defined. Strains of the human gut commensal *Bifidobacterium longum* subsp. *longum* utilise arabinose- and xylose-containing glycans, which are found in the pectin and hemicellulose layers of plant cell walls. To gain insight into the metabolism of these two pentoses as well as ribose, a naturally occurring sugar and a component of RNA and ATP, we identified and analysed the genes responsible for their uptake and subsequent catabolism. Based on transcriptomic data and mutant phenotype analyses, we show that these three pentoses share a common, ABC-type uptake system encoded by *penABCD*. Furthermore, we identify a gene cluster, *araBDA*, and two genes, *xylA* and *xylB*, that are required for conversion of arabinose and xylose, respectively, into xylulose-5-phosphate, and *rbsK*, which converts ribose into ribose-5-phosphate. These intermediate metabolic products enter the bifid shunt, an energy-generating fermentative pathway typical of bifidobacteria. We also show that arabinose and xylose are co-metabolized, while xylose is preferentially utilised before ribose. This study provides molecular insights using a multi-omics approach, including comparative genomics and transcriptomics combined with mutational analysis, into how *B. longum* subsp. *longum* metabolizes pentose-containing plant glycans, common yet indigestible components of the adult human diet.

## Introduction

Bifidobacteria are saccharolytic anaerobic Gram-positive bacteria with an unusual, bifurcated morphology, commonly isolated from fecal samples of various mammals including humans^1-3^ Multiple bifidobacterial species can be found in the gastrointestinal tract of a given individual, with species abundance dependent on multiple factors, particularly host diet.^4^ Human-derived bifidobacteria typically encode saccharolytic activities that enable them to utilize a wide variety of carbohydrates, including certain dietary fibers. The broad saccharolytic nature of bifidobacterial metabolism is also reflected in their genomes, with 8%-12% of their coding content dedicated to carbohydrate utilization.^5-7^ This metabolism results in the production of lactate and acetate, as well as other metabolites that inhibit growth of pathogens in the human gut and that positively modulate the immune system,^8-11^ motivating the use of various bifidobacterial strains as probiotics.

Members of the *Bifidobacterium longum* species are among the most frequently encountered bifidobacteria in the human gut, with *B. longum* subsp. *infantis* and subsp. *iuvenis* commonly found in infants.^12^ Their presence in infants has been associated with the ability to utilize human milk oligosaccharides, whereas the higher prevalence of *B. longum* subsp. *longum* in adults versus infants may be due to its ability to utilize particular plant-derived glycans present in the adult diet.^7^ Aside from cellulose, plant-derived foods typically contain two dominant glycan-rich components, hemicellulose and pectin. Hemicellulose represents part of the cellulose matrix of the plant cell wall that is responsible for its rigidity, while pectin is responsible for the slippage, deposition, and extension of the cellulose matrix and the adhesive material between plant cells (Supplemental Figure S1).^13^^,^^14^ Specific polysaccharides that make up these hemicellulose and pectin layers include arabinan, arabinoxylan, and xylan, which are polysaccharides containing the pentose sugars L-arabinose (hereafter, arabinose) and D-xylose (xylose). Concordantly, it was previously shown that *B. longum* subsp. *longum* generally grows well on arabinose and xylose.^15^^,^^16^ Moreover, *B. longum* subsp. *longum* has been reported to utilize a third pentose sugar, D-ribose (ribose).^17^ Ribose can be derived from dietary intake (like arabinose and xylose) from plants or animals as well as from RNA hydrolysis due to natural turnover of human cells lining the gut or of deceased gut microbes (e.g., due to bacteriophage attack or antimicrobial exposure).^18^ Thus, mechanistic understanding of how *B. longum* subsp. *longum* metabolizes these pentose sugars will provide critical insight into the strategies that enable its colonization and persistence in the adult gut, and more generally into how diet shapes gut microbiome composition and function.

Various gut commensals, including *Escherichia coli*, are able to utilize pentoses, leading to considerable knowledge pertaining to the genes involved in their metabolism. *E. coli* cells import arabinose using two distinct systems, a high-affinity ABC-type transporter system encoded by *araFGH*, and an alternative, low-affinity transporter belonging to the sugar-proton symporter family encoded by *araE.*^19^ Following internalization of arabinose, the products of the *araBDA* gene cluster convert arabinose to xylulose-5-phosphate, which then enters the pentose-phosphate pathway.^20^^,^^21^ Xylose is imported into *E. coli* by a high-affinity ABC-type transporter system encoded by *xylFGH* or by the low-affinity AraE.^22^ After xylose is imported, XylA and XylB convert xylose into xylulose-5-phosphate.^23^ In *E. coli*, the *rbsDACBK* gene cluster is responsible for utilization of ribose.^24^ This cluster encodes a ribose-specific ABC-type uptake system, RbsACB*;* a ribokinase, RbsK, responsible for conversion of ribose into D-ribose 5-phosphate; and a ribose mutarotase, RbsD, responsible for conversion between the *α*-furanose and *β*-furanose forms of ribose, the latter of which is used by RbsK. Furthermore, several additional low-affinity transporters of ribose are encoded by the *E. coli* genome, likely as alternative uptake systems.^25^

While these pentose metabolic pathways have been experimentally established in *E. coli* based on biochemical validation, corresponding functional genetic information for *B. longum* subsp. *longum* is fragmented, somewhat contradictory and largely based on *in silico* analyzes (arabinose uptake and metabolism by AraFGH and AraBDA^26^; xylose uptake and metabolism by XylFGH^27^ and XylAB,^23^^,^^28^) combined with transcriptomics and *in vitro* promoter binding studies (ABC-type transporter system *fruABCD*^17^^,^^28^ and homologs *rbsEKFG*^17^ binding to fructose, xylose and ribose). These findings suggest that pentose metabolism in *B. longum* subsp. *longum* may rely on as yet unrecognized or unverified transport and regulatory pathways whose clarification is important for understanding energy yield and metabolic outputs in this key gut commensal. To achieve a comprehensive and experimentally corroborated understanding of pentose metabolism by *B. longum* subsp*. longum*, we genetically and biochemically dissected pentose utilization in strain NCIMB 8809 as a prototype. Our findings have broad implications for dietary fiber utilization, microbiome ecology, and host–microbe interactions in the human gut and provide a foundation for rational manipulation of bifidobacteria as probiotics.

## Materials and methods

### Strains, cultivation conditions, and media

All strains and plasmids used in this study are listed in Table S1. *B. longum* subsp. *longum* strains were routinely cultivated under anaerobic conditions at 37 °C in modified de Man Rogosa Sharpe (mMRS^29^; medium excluding a carbohydrate source and then supplemented with 0.5% lactose (weight/volume [w/v]; Sigma-Aldrich, Germany) and 0.06% cysteine-HCl (w/v; Sigma-Aldrich). Bifidobacterial strains were incubated anaerobically in a modular atmosphere-controlled system (Davidson and Hardy, Belfast, United Kingdom) at 37 °C. *E. coli* strains were routinely cultivated in Difco™ LB (Luria Bertani) broth (BD, UK) at 37 °C with agitation at 180 rpm. Where appropriate, the growth medium was supplemented with kanamycin (Km; 100 μg ml^−1^ for *E. coli*), erythromycin (Erm; 250 μg ml^−1^ for *E. coli*, 100 μg ml^−1^ for *B. longum*) or tetracycline (Tc; 10 μg ml^−1^ for *E. coli*, 5 μg ml^−1^ for *B. longum*).

### Growth measurements

Carbohydrate utilization by bifidobacterial strains was examined in mMRS medium supplemented with cysteine-HCl (0.06% w/v; Sigma-Aldrich, Germany) and a particular carbohydrate (0.5%, w/v). Assessed carbohydrates were lactose (Sigma-Aldrich, Germany), arabinose (Sigma-Aldrich, Germany), xylose (Sigma-Aldrich, Germany), ribose (Merck, Germany), cytidine (Carbosynth, UK), inosine (Sigma-Aldrich, Germany), and uridine (Sigma-Aldrich, Germany). To quantify bacterial growth dynamics and final optical density, 5 ml of freshly prepared mMRS medium supplemented with a particular carbohydrate was inoculated with 50 μl of an overnight culture grown (diluted to OD_600 nm_~1) of an overnight culture grown in mMRS + 0.06% cysteine-HCl (w/v) + 0.5% lactose (w/v). Uninoculated mMRS medium and/or medium without supplemented sugar was used as negative controls. Cultures were incubated anaerobically at 37 °C and the OD_600 nm_ was measured after 24 h using a spectrophotometer. All growth data in this study are shown as the mean ± 1 SD of at least three biological replicates. Comparisons between two groups were performed using a Student’s t-test. Fisher's exact test was used to determine if a genetic trait is independent of strain origin.

### HPAEC-PAD analysis of carbohydrate content in growth medium

*B. longum* subsp. *longum* NCIMB 8809 was grown in mMRS supplemented with 0.06% cysteine-HCl and a combination of (i) 0.25% (w/v) lactose and 0.25% (w/v) glucose; (ii) 0.25% (w/v) glucose and 0.25% (w/v) arabinose; (iii) 0.25% (w/v) arabinose and 0.25% (w/v) xylose; or (iv) 0.25% (w/v) xylose and 0.25% (w/v) ribose. Aliquots were taken at time points 0 h, 2 h, 3 h, 4 h, 5 h, 6 h, 7 h, 8 h, 9 h, 10 h, and 24 h, and then analyzed via High-Performance Anion Exchange Chromatography–Pulsed Amperometric Detection (HPAEC-PAD; Thermo Scientific Dionex, Sunnyvale, CA, ICS-3000 system). Carbohydrate fractions from the growth curves mentioned above were separated on a CarboPac PA1 analytical exchange column (250 mm × 4 mm) with a CarboPac PA1 guard column (50 mm × 4 mm) and visualized using a pulsed electrochemical detector (ED40) in PAD mode (Thermo Scientific Dionex). Elution was performed with 100 mM of potassium hydroxide (KOH) at a constant flow rate of 0.063 ml/min at 30 °C for 7.5 min. Chromatographic profiles of each of the carbohydrates tested were used as reference standards for identification during quantitative analyzes of the remaining carbohydrate in the supernatants. Chromeleon software v7.3 (Dionex Corporation) was used for integration and evaluation of the obtained chromatograms.

### Transcriptome analysis using RNA-seq

*B. longum* subsp. *longum* NCIMB 8809 was grown overnight in mMRS + 0.06% cysteine-HCl + 0.5% (w/v) lactose and then 100 µl of the overnight culture was subcultured in 10 ml of mMRS + 0.06% cysteine-HCl + 0.5% (w/v) lactose, arabinose, xylose or ribose until an OD_600 nm_ of 0.5–0.8, which in all carbohydrate combinations was shown to correspond to the mid-log phase as based on previously obtained growth curves (Supplemental Figure S2). Four milliliters of each culture were collected via centrifugation (Dlab D3024R) at 5500*g* for 4 min. Harvested cells were resuspended in DNA/RNA shield (Zymo Research, CA, USA) and stored at –80 °C. For all samples and each test condition biological duplicates were prepared (Table S2). The frozen cell suspension samples were then sent on dry ice to the University of Groningen for RNA extraction, rRNA depletion, library construction, and RNA sequencing. To eliminate ribosomal RNA from the 250 ng total RNA, the RiboCop rRNA depletion kit (Lexogen, Vienna, Austria) was employed. Subsequently, the NEBNext Ultra II Directional RNA Library Prep Kit (E7765, New England Biolabs) was utilized to prepare the library preps for Illumina sequencing.

The sequencing process was performed on an Illumina NextSeq 1000, generating 100-bp single-end reads with an average read depth of 8–12 million reads per sample. The quality of the resulting fastq reads was assessed using FastQC v0.11.9 (Babraham Bioinformatics, Cambridge), followed by mapping on the reference genome using Bowtie2 v2.4.2 with default settings. The resulting SAM files were converted to BAM format using SAMtools v1.11, and gene counts were obtained using featuresCounts v2.0.1 (Subread/2.0.2). Feature counts from all samples were combined into a single count matrix and normalized using the median-of-ratios method implemented in DESeq2.^30^ The normalized count data were used as input for the T-REx2 framework^31^ where differential gene expression analysis was performed. Results were visualized using standard approaches, including volcano plots and principal component analysis (PCA). The resulting reads were further visualized using IGV-web.^32^ Homologs of the identified metabolic genes were identified using tBlastn analysis^33^ or 90 *B. longum* subsp. *longum* strains isolated in the MicrobeMom study together with 46 publicly available strains on NCBI (using an 80% aa identify across 95% coverage cut off, E-value: 1e-20).

### Generation of insertion mutants

Individual insertion mutations of eight genes (*araD*, *araA*, *penA*, *penB*, *xylA*, *xylB*, *rbsK* and *araG*; corresponding locus tags can be found in Table S3) in the genome of *B. longum* subsp. *longum* NCIMB 8809 were generated as follows: an internal fragment of each gene to be targeted was amplified by PCR (Q5 High-Fidelity DNA polymerase) using chromosomal DNA of *B. longum* subsp. *longum* NCIMB 8809 as template (details about amplicon length and position and associated primer sequences are listed in Table S2). The generated PCR product for each intended insertion mutation was then ligated to pFREM2^34^^,^^35^ using HindIII and XbaI (New England Biolabs (NEB), Ipswich, MA, USA) and introduced into *E. coli* EC101 as a cloning host.^36^ *E. coli* EC101 derivatives containing the recombinant pFREM2 constructs were selected on LB agar containing Erm. After verification of these pFREM2 derivatives via sequencing (Genewiz; Leipzig, Germany), the resulting recombinant plasmids (pFREM2:*araD*, pFREM2:*araA*, pFREM2:*penA*, pFREM2:*penB*, pFREM2:*xylA*, pFREM2:*xylB,* pFREM2:*rbsK* and pFREM2:*araG*) were extracted using a GeneJET Plasmid Maxiprep kit (Fisher Scientific; Waltham, MA, USA). *B. longum* subsp. *longum* NCIMB 8809 was transformed as previously described.^35^^,^^37^^,^^38^ To validate their genetic integrity, individual representatives of each intended insertion mutant (NCIMB8809-Δ*araD*, NCIMB8809-Δ*araA*, NCIMB8809-Δ*penA*, NCIMB8809-Δ*penD*, NCIMB8809-Δ*xylA*, NCIMB8809-Δ*xylB,* NCIMB8809-Δ*rbsK* and NCIMB8809-Δ*araG*) were verified by PCR in which the forward primer targeted the genome of *B. longum* subsp. *longum* NCIMB 8809 close to the expected plasmid insertion point and the reverse primer targeted the (chromosomally inserted) plasmid (OneTaq 2xMM; NEB). Each insertion mutant was verified by DNA sequencing of the corresponding PCR product (Genewiz).

### Construction of recombinant plasmids for genetic and phenotypic complementation

For complementation of a given insertion mutant, the gene of interest was PCR amplified to generate an amplicon that included its presumed native promoter when possible (i.e. when the promoter was located immediately upstream of the gene; details on relevant gene locus tag, location, primers, and promoters in Table S4). PCR products were ligated to pBM5, a derivative of pAM5 with a Type I restriction-modification site removed,^35^^,^^39^ using MfeI and NheI restriction sites, and introduced into chemically competent *E. coli* DH5α cells as the cloning host. The individual recombinant plasmids were then transferred into EC101 cells harboring plasmid pNZEM, which expresses a Type II methylase from strain NCIMB 8809 to allow methylation to bypass the most active restriction-modification system.^40^ For complementation involving genes requiring an artificial promoter, the p44 promoter region of pNZ44^41^ was cloned into pBM5 directly upstream of the gene of interest. Constructs were verified via DNA sequencing (Plamidsaurus, Eugene, OR, USA). Plasmid DNA was extracted (GeneJET Plasmid Miniprep kit; Thermo Fisher Scientific, USA) and introduced into the corresponding *B. longum* subsp. *longum* NCIMB 8809-derived mutant by electroporation. Plasmid presence in transformants was verified by colony PCR using primers that target the MCS site of the plasmid, including the complementing gene (OneTaq 2xMM; NEB).

### Growth measurements of *B. longum* subsp. *longum* NCIMB 8809 strains

Growth profiles of *B. longum* subsp. *longum* NCIMB 8809 wild-type and mutant/complemented strains were measured in mMRS medium using 0.5% (w/v) lactose, arabinose, xylose, ribose, fructose, or galactose as the sole carbohydrate source. Five milliliters of freshly prepared mMRS medium with a particular carbohydrate were inoculated with 50 μl of a *B. longum* subsp. *longum* overnight culture (OD_600 nm_ ~ 1) grown in mMRS + 0.06% (w/v) cysteine-HCl + 0.5% (w/v) lactose. Uninoculated mMRS medium and mMRS without carbohydrate were used as negative controls. Cultures were incubated anaerobically at 37 °C and OD_600 nm_ was measured after 24 h using a spectrophotometer. Assays were repeated in triplicate. To determine growth rates, strains were grown in mMRS + 0.06% (w/v) cysteine-HCl using 0.2% (w/v), 0.5% (w/v), 2% (w/v), or 4% (w/v) arabinose or 0.5% (w/v) lactose under anaerobic conditions with hourly measurements of OD_600 nm_. Growth rates (h^−1^) were calculated from OD_600 nm_ values obtained between 3 h and 9 h.

### Overexpression and purification of His-tagged AraB, AraD, AraA, XylA, XylB, and xylulose-5-phosphate/fructose-5-phosphate phosphoketolase

For overexpression and purification of the six His-tagged proteins (designated here as AraB_His_, AraD_His_, AraA_His_, XylA_His_, XylB_His_, and the xylulose-5-phosphate/fructose-5-phosphate phosphoketolase (XPPKT_His_)), plasmids pET28b: *araA*, pET28b: *araB*, pET28b: *araD*, pET28b: *xylA*, pET28b: *xylB*, and pET28b: *xppkt*, respectively, were generated as follows (details regarding recombinant plasmids, locus tags, position in genome, and primers in Table S5). The respective genes were amplified via PCR (Q5 High-Fidelity DNA polymerase, NEB) using genomic DNA extracted from *B. longum* subsp. *longum* NCIMB 8809 as a template and cloned into pET28b, utilizing its encoded N-terminal His-tag (Novagen), with NheI and NotI. *E. coli* 10-beta cells (NEB) were used as the cloning host,^36^ before transferring the individual recombinant plasmids into BL21 (DE3) cells. All constructs were verified via DNA sequencing (Plasmidsaurus, Eugene, OR, USA).

For protein overexpression, 100 ml of NZY Auto-Induction LB media (1% (v/v), NZYtech, Lisbon, Portugal) were inoculated with 1 ml of an overnight culture of a particular *E. coli* BL21 (DE3)-derivative strain grown in LB media and incubated for 24 h at 24 °C with shaking at 300 rpm. Cells were harvested via centrifugation (Thermo Scientific SL16R) before resuspension in a lysis buffer (50 mM Tris-Base (Fisher Bioreagents, USA), 300 mM NaCl (Fisher Bioreagents), 50 mM CaCl_2_ (Fisher Bioreagents), 10 mM imidazole (Sigma-Aldrich)). Cell lysis was achieved using a mini-beadbeater (BiospecProducts, USA; 3 × 30 s alternating with 30-s incubations at 4 °C). Protein purification was performed via affinity chromatography using Ni-NTA columns (Qiagen, UK), using elution buffers with imidazole concentrations ranging from 100 mM to 500 mM. To assess the molecular weight of the recombinant AraB_His_, AraD_His_, AraA_His_, XylA_His_, XylB_His_, and XPPKT_His_ proteins, sodium dodecyl sulfate-polyacrylamide gel electrophoresis (SDS-PAGE) was performed as previously described ^42^ including Color Prestained Protein Standard, Broad Range (10–250 kDa, NEB), and gels were stained using Coomassie Brilliant Blue. Protein concentrations were estimated using a Qubit® Fluorometer (Thermo Scientific, Gloucester, UK) and the corresponding protein assay kit (Thermo Scientific).

### Enzyme activity testing based on acetyl-phosphate release using hydroxamate assays

To investigate the activity of the enzymes encoded by the *araBDA* gene cluster for metabolism of arabinose into xylulose-5-phosphate and the activity of XylA and XylB for metabolism of xylose into xylulose-5-phosphate, various combinations of the enzymes were incubated for 16 h in 0.5 mM thiamine pyrophosphate (TPP; Sigma), 1 mM DTT (Sigma), 5 mM MgCl_2_ (Merck), 16.43 mM adenosine triphosphate (ATP; Sigma), and 50 mM morpholineethanesulfonic acid (MES) buffer (pH 6.5; Sigma), with 1 mM XPPKT_His_ added to convert D-xylulose-phosphate into acetyl-phosphate (Figure 5). Furthermore, to determine the function of each enzyme, various enzyme-substrate combinations were used to start the enzymatic reactions at intermediate steps in the respective pathways. The released acetyl-phosphate was measured using a hydroxamate assay.^43^^,^^44^ 100 microliters of 2 M hydroxylamine hydrochloride (pH 6.5) were added to 100 μl of reaction mixtures and incubated at room temperature for 30 min, followed by the addition of 50 μl of 15% (w/v) trichloroacetic acid, 75 μl of 4 M hydrochloric acid, and 75 μl of 5% (w/v) iron chloride in 0.1 M HCl. Color change was measured using standard reaction mixtures including the respective substrate with only added XPPKT_His_ as a blank. During the assay, released acetyl-phosphate binds with hydroxylamine to form hydroxamic acid, which further interacts with trivalent iron to form a brown/reddish complex, which can be measured using a spectrometer at a wavelength of 505 nm^43^. All essays were performed in triplicate, data is shown as the mean ± 1 SD shown, while a Student’s t-test was used for comparisons.

## Results

### *B. longum* subsp. *longum* strains exhibit variable capacity to grow on ribose

**Figure 1.**
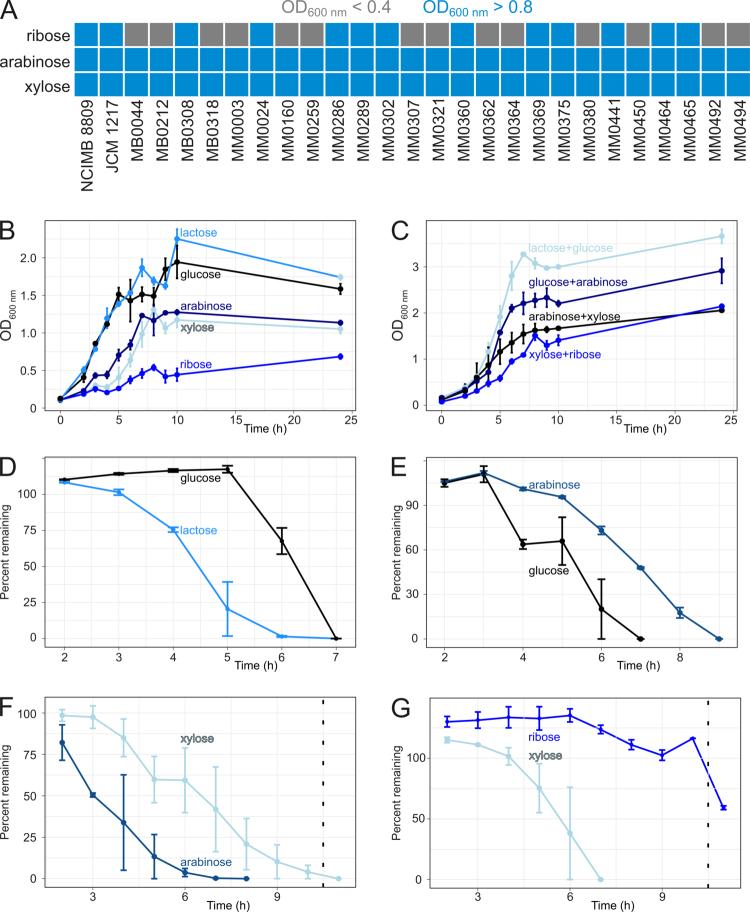
*Bifidobacterium longum* subsp. *longum* strains exhibit variable capability for growth on ribose and hierarchy of carbohydrate source utilization. Cultures were grown for 24 h in mMRS containing 0.25% (w/v) arabinose, xylose, or ribose. Ability to grow was assessed based on optical density at 600 nm (OD_600 nm_ readings; blue: growth, OD_600 nm_ > 0.8; gray: no growth, OD_600 nm_ > 0.4). (A) Only 13 of 27 strains were able to grow on ribose, whereas all were able to grow on arabinose or xylose (*n* = 3). (B) Growth curves of *B. longum* subsp. *longum* NCIMB 8809 in mMRS supplemented with 0.25% (w/v) arabinose, glucose, lactose, ribose, or xylose (*n* = 4). (C) Growth curves in combinations of the carbohydrate sources in B. (D) HPAEC-PAD analyzes of supernatants revealed that glucose was only utilized once lactose was almost fully depleted during growth in 0.25% (w/v) lactose + 0.25% (w/v) glucose (*n* = 4). (E) Glucose was utilized before arabinose during growth in 0.25% (w/v) glucose + 0.25% (w/v) arabinose (*n* = 4). (F) Arabinose was utilized before xylose during growth in 0.25% (w/v) arabinose + 0.25% (w/v) xylose (*n* = 4). (G) Ribose was only utilized once xylose was almost fully depleted during growth in 0.25% (w/v) Xyl + 0.25% (w/v) ribose (*n* = 4).

With the overall goal to analyze pentose sugar metabolism in *B. longum* subsp. *longum*, we tested the ability of a panel of 27 *B. longum* subsp. *longum* strains to grow on arabinose, xylose, or ribose. The panel consists of 25 strains that were previously isolated from either infant or adult fecal samples of mother-infant dyads as part of the MicrobeMom study,^45^^,^^46^ as well as two commonly used strains (NCIMB 8809, isolated form infant stool, and JCM 1217, isolated from adult stool; Figure 1). As a negative control of this carbohydrate-dependent growth analysis, none of the strains was able to exhibit appreciable growth in mMRS medium without the addition of a fermentable carbohydrate source (OD600nm < 0.1). All 27 strains were able to grow on mMRS supplemented with either arabinose or xylose, reaching OD_600 nm_ values > 0.8 after 24 h of cultivation, indicating that arabinose/xylose metabolism is a common trait of this species irrespective of the isolation source, in agreement with previous studies.^15-17^ In contrast, with ribose as the sole carbohydrate source, only 12 of the 27 strains exhibited appreciable growth (OD_600 nm_ > 0.8 after 24 h), indicating that the ability to utilize this pentose sugar is not conserved among the *B. longum* subsp. *longum* subspecies. The ability to utilize ribose was independent of whether the strain originated from infant or adult (Figure 1; Fisher's exact test: *p* = 1). Seven strains were also tested individually for their ability to utilize either of three (ribose-containing) nucleosides (cytidine, inosine, or uridine); however, none of them showed appreciable growth on either of these nucleosides (Supplemental Figure S3).

## Analysis of substrate preferences reveals utilization hierarchy

To investigate preferential pentose sugar utilization in *B. longum* subsp. *longum*, we focused on *B. longum* subsp. *longum* NCIMB 8809 since this strain exhibits growth on all three pentose sugars and was previously shown to be genetically amenable.^35^^,^^40^ Following incubation of *B. longum* subsp. *longum* NCIMB 8809 for 24 h on mMRS supplemented with 0.25% (w/v) arabinose, xylose, or ribose, NCIMB 8809 fully utilized the arabinose within 7 h (Supplemental Figure S4A) and the xylose within 5 h (Supplemental Figure S4B). However, the strain did not fully utilize all available ribose; HPAEC-PAD analysis of samples taken after 24 h of cultivation demonstrated that ~20% of the ribose remained (Supplemental Figure S4C,D). This finding is consistent with the faster growth rate for this strain on mMRS containing arabinose (0.15 h^−1^, Figure 1B) and xylose (0.14 h^−1^, Figure 1B) compared to ribose (0.03 h^−1^, Figure 1B). To assess if NCIMB 8809 exhibits preferential carbohydrate utilization, we grew it in mMRS containing one of four carbohydrate combinations (0.25% (w/v) of each sugar; Figure 1C): (i) lactose and glucose; (ii) glucose and arabinose; (iii) arabinose and xylose; or (iv) xylose and ribose. The strain is able to utilize the combination of lactose and glucose the quickest (0.39 h^−1^, Figure 1C), before glucose and arabinose (0.30 h^−1^, Figure 1C), arabinose and xylose (0.24 h^−1^, Figure 1C), and xylose and ribose (0.16 h^−1^, Figure 1C). When grown on lactose and glucose, lactose was fully utilized before glucose was used for growth (Figures 1D, S2E). This result is in agreement with a previous study that showed a preference for lactose over glucose in *B. longum* subsp. *longum* NCC2705,^47^ suggesting that this preference may be general across the subspecies. When arabinose and glucose were both present, NCIMB 8809 co-utilized these sugars (Figures 1E, S2F), although it appears that glucose is initially preferred, as arabinose is utilized at a slower rate until glucose levels are completely depleted. A similar pattern can be seen for arabinose and xylose, where arabinose is utilized faster until the percentage remaining was ~50% (Figures 1F, S4G). In contrast, NCIMB 8809 fully metabolized xylose before using ribose for growth (Figures 1F, S4H). These findings indicate that NCIMB 8809 employs a multi-tier substrate utilization hierarchy.

### Transcriptome analysis reveals genes important for pentose utilization

**Figure 2.**
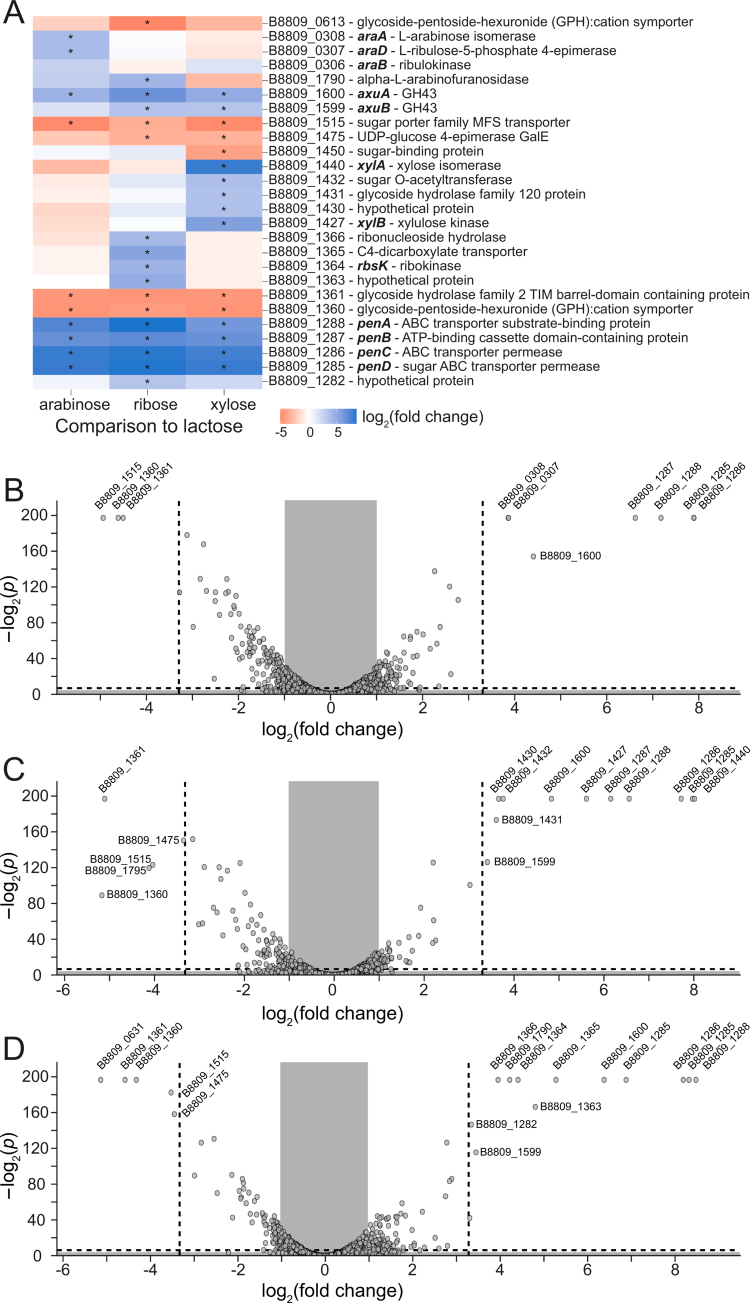
Differential expression analysis reveals key genetic pathways for carbohydrate source utilization. (A) Relative expression of selected genes in *B. longum* subsp. *longum* NCIMB 8809 during growth in arabinose, xylose, and ribose compared to lactose (*n* = 2). Significantly differentially expressed (–log_2_(*p*)>5 and |log_2_(fold change)|>3) are highlighted with asterisks. (B–D) Differential expression of all genes in arabinose (B), xylose (C), and ribose (D) compared to lactose. Dashed lines represent the thresholds used to define significance in (A). A limit of 200 was set for the −log2(*p*) values. The sphere around dots represents the expression level of the gene.

**Figure 3.**
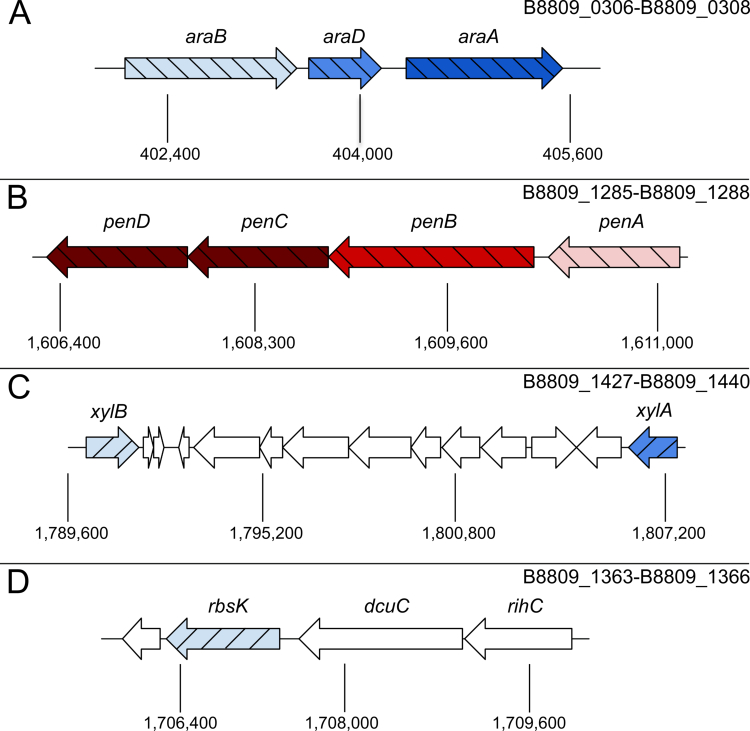
Gene clusters involved in utilization of arabinose, xylose, and ribose. (A) *araBDA*_*8809*_ is responsible for conversion of arabinose to xylulose 5-phosphate. (B) The ABC uptake system *penABCD* is responsible for import of arabinose, xylose, and ribose. (C) *xylA*_*8809*_ and *xylB*_*8809*_ are responsible for conversion of xylose into xylulose and xylulose 5-phosphate, respectively. (D) The gene cluster containing *rbsK*_*8809*_ is responsible for conversion of ribose into D-ribulose-5-phosphate. Light blue: kinase; blue: epimerase; dark blue: isomerase; light red: substrate binding protein; red: ATP-binding protein; dark red: ABC transporter permease.

To investigate the assimilation pathways and identify genes important for the utilization of each pentose sugar, we performed RNA-seq analysis of *B. longum* subsp. *longum* NCIMB 8809 grown in 0.5% (w/v) arabinose, xylose, or ribose and compared to growth in 0.5% (w/v) lactose (Figure 2). Among the genes that were differentially expressed in arabinose compared with lactose (Figure 2A,B), we identified a cluster of three adjacent genes, locus tags B8809_306, B8809_307, and B8809_308 (designated here as *araBDA*_*8809*_, Figure 3A), which are predicted to encode a kinase, sugar epimerase and isomerase, respectively. Based on the transcript mapping, *araD*_*8809*_ and *araA*_*8809*_ constitute an operon, potentially together with *araB*_*8809*_ (Supplemental Figure S5). This cluster is homologous and syntenic to *araBDA* of *E. coli*, which is responsible for the conversion of arabinose to xylulose-5-phosphate (sequence similarities are listed in Table S5). Furthermore, a cluster of four adjacent genes, within a single operon (Supplemental Figure S5) with locus tags B8809_1285–B8809_1288 (designated here as *penABCD*, Figure 3B), also exhibited an arabinose-dependent increase in transcription. This cluster is predicted by BlastP to encode a sugar binding protein and an associated ABC-type transporter system.

Genes at two locations were significantly upregulated (−log_2_(*p*)>80) when grown on xylose compared to lactose (Figure 2A,C). The first xylose-induced cluster consists of *penABCD*, the cluster also upregulated in the presence of arabinose (Figure 2A,C). The second xylose-induced cluster (Figure 3C) includes four genes predicted to encode an intracellular glycoside hydrolase (GH) from the GH120 family (B8809_1431), a hypothetical protein (B8809_1430), a sugar kinase (B8809_1427, designated here as *xylA*_*8809*_) and a sugar epimerase (B8809_1440, designated here as *xylB*_*8809*_), located in three transcriptional units (Supplemental Figure S5C). Sequence similarities can be found in Table S5.

Two gene clusters were significantly upregulated during growth in ribose compared to lactose (Figure 2A,D). Along with *penABCD*, a set of four adjacent genes (Figure 3D) predicted to encode a ribokinase (B8809_1364, designated here as *rbsK*_*8809*_), a transmembrane ion transporter (B8809_1365, similar to *dcuC* in *E. coli*), a ribonucleoside hydrolase (B8809_1366, similar to *rihC* in *E. coli*), and a hypothetical protein (B8809_1363) were upregulated, although it is unclear if they constitute a single or multiple transcriptional units (Supplemental Figure S5D). Homology and predicted functions can be found in Table S6.

Two genes, each encoding an extracellular arabinofuranosidase (GH43 family), were shown to exhibit increased expression during growth in arabinose, xylose, or ribose compared to lactose. These genes correspond to locus tags B8809_1599 (expression increased significantly when grown in a medium with xylose and ribose, but not with arabinose) and B8809_1600 (increased expression when grown in medium containing any of the three pentoses), which we previously had shown to be required for growth of *B. longum* subsp. *longum* on cereal-derived arabinoxylan.^35^ Expression of a third gene predicted to encode a GH43 family member (locus tag B8809_1790) was significantly higher during growth in ribose compared to lactose, and the protein product of this gene is 99% similar to the previously characterized *α*-arabinofuranosidase *Bl*ArafA (BLLJ_1854), which cleaves *α*-1, 3-arabinose side chains from arabinogalactan.^48^

Furthermore, two genes, each predicted to encode an MFS transporter (locus tags B8809_1515 and B8809_1360), were significantly downregulated during growth in arabinose, xylose, and ribose compared to lactose. A third gene, also predicted to encode an MFS transporter (B8809_0613), was significantly downregulated in ribose. The decrease in B8809_1360 expression is consistent with its predicted function in importing lactose and galactose (74.73% identity to *lacS* in *B. breve*^49^). The product of B8809_1515 is predicted to import glucose (it exhibits 98.71% identity with *glcP*, which encodes an MFS transporter in *B. longum* subsp. *longum* NCC2705^47^).

These transcriptome-guided assignments provided the basis for subsequent genetic validation of both conserved and previously unrecognized gene clusters that contribute to pentose utilization in *B. longum* subsp. *longum*.

### Phenotypic validation of pentose metabolism genes via mutational analysis

**Figure 4.**
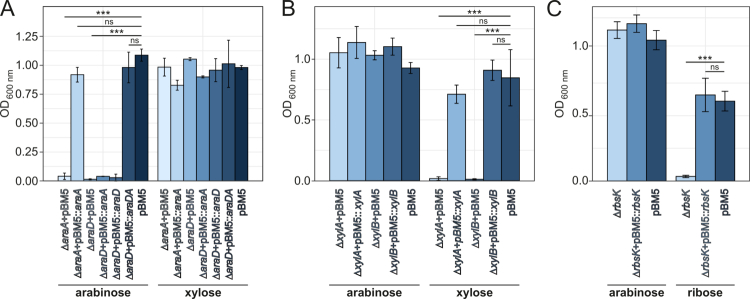
The *araBDA*_*8809*_, *xylAB*_*8809*_, and *rbsK*_*8809*_ gene clusters or genes are involved in growth in arabinose, xylose, and ribose, respectively. Shown are OD_600 nm_ values after 24 h of anaerobic growth in mMRS supplemented with the carbohydrate sources shown (*n* = 3). (A) *araA*_*8809*_ and *araD*_*8809*_ are required for growth in 0.5% (w/v) arabinose but not xylose. (B) *xylA*_*8809*_ and *xylB*_*8809*_ are required for growth in 0.5% (w/v) xylose but not arabinose. (C) *rbsK* is required for growth in 0.5% (w/v) ribose but not arabinose. ***: *p* < 0.0005, **: *p* < 0.005, ns: no significance.

To validate the involvement of *araABD*_*8809*_ in arabinose metabolism, we constructed individual insertion mutants in *araA*_*8809*_ and *araD*_*8809*_. Disruption of either gene resulted in complete loss of growth on arabinose as the sole carbohydrate source, demonstrating the essential role of this cluster in arabinose metabolism (Figure 4A). Introduction of plasmid pBM5:*araD* into NCIMB 8809-Δ*araD* failed to restore the ability to grow in arabinose-containing medium, likely reflecting a polar effect of the mutation on the downstream *araA*_*8809*_. Indeed, introduction of both *araD*_*8809*_ and *araA*_*8809*_ on a single plasmid (pBM5:*araA*+*araD*) restored growth in arabinose, resulting in a final OD_600 nm_ > 1 (Figure 4A). These results indicate that, as expected, *araA*_*8809*_ and *araD*_*8809*_ are both required for arabinose metabolism. Complementation of growth in arabinose was also observed following introduction of plasmid pBM5:*araA* into NCIMB 8809-Δ*araA*.

To validate the involvement of the kinase- (*xylA*_*8809*_) and epimerase- (*xylB*_*8809*_) encoding genes in xylose metabolism, we generated insertion mutants in each of the genes (to generate strains NCIMB 8809-Δ*xylA* and NCIMB 8809-Δ*xylB*, respectively). When grown in mMRS with xylose as the sole carbohydrate source, neither mutant reached an OD_600 nm_ > 0.1 (Figure 4B), while growth in arabinose was not affected. Introduction of plasmid pBM5:*xylA* into NCIMB 8809-Δ*xylA* restored utilization of xylose to a similar level as wild type harboring an empty plasmid (Figure 4B). Similar complementation was observed for introduction of plasmid pBM5:*xylB* into NCIMB 8809-Δ*xylB* (Figure 4B).

To validate the involvement of *rbsK*_*8809*_ in ribose metabolism, we constructed insertion mutant NCIMB 8809-Δ*rbsK*. This mutant exhibited significantly impaired growth in ribose compared to wild type (*p* < 0.005, Figure 4C), while growth in arabinose was unaffected. When plasmid pBM5:*rbsK* was introduced into NCIMB 8809-Δ*rbsK*, growth in ribose reached a final OD_600 nm_ similar to that of the wild type harboring an empty pBM5 (Figure 4C).

Taken together, these findings confirm the functional significance of our transcriptomic analyzes.

### Biochemical analyzes of arabinose and xylose metabolic enzymes

**Figure 5.**
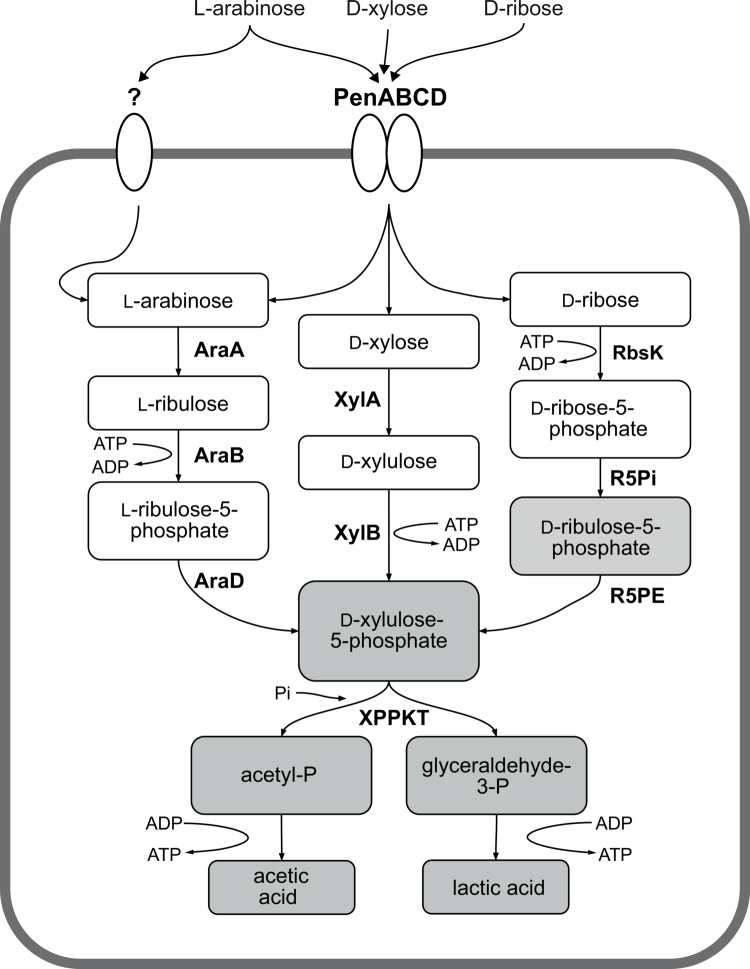
Schematic of arabinose, xylose, and ribose degradation through the “bifid shunt” in bifidobacteria. Abbreviations: PenABCD, ATP transporter for arabinose, xylose, and ribose; AraA, arabinose isomerase; AraB, ribulose-5-phosphate epimerase (L-configuration); AraD, ribulokinase; XylA, xylose isomerase; XylB, xylulose kinase; XPPKT, xylulose-5-phosphate/fructose-6-phosphate; RbsK, ribokinase; R5Pi, ribose-5-phosphate isomerase; R5PE, ribulose-5-phosphate epimerase (D-configuration). Gray boxes are part of the bifid shunt but are not fully displayed.

To biochemically confirm the predicted enzymatic activities of AraA_8809_, AraB_8809_, and AraD_8809_ in the arabinose metabolic pathway, each gene was cloned into the expression vector pET28b and used for overexpression and purification of the respective proteins in *E. coli* (see for details Materials and Methods). The final product of the AraABD- or XylAB-catalyzed pathway is xylulose-5-phosphate, which is further converted into acetyl-phosphate and glyceraldehyde-3-phosphate by XPPKT (Figure 5). The released acetyl-phosphate can be measured using a colourimetric assay, in which acetyl-phosphate binds to hydroxylamine leading to hydroxamic acid. Activity of the XPPKT_8809His_ enzyme and subsequent assessment of acetyl-phosphate was confirmed by the addition of 4.35 mM xylulose-5-phosphate in the reaction mix, releasing 3.95 ± 0.21 mM of acetyl-phosphate. Purified AraA_8809His_, AraB_8809His_, and AraD_8809His_ were then tested for their ability to convert arabinose to xylulose-5-phosphate using hydroxamate assays to measure released acetyl phosphate. Different enzyme combinations and intermediate metabolites were used to confirm the role of each of the recombinant enzymes in catalyzing specific steps of the arabinose metabolic pathway.

**Figure 6.**
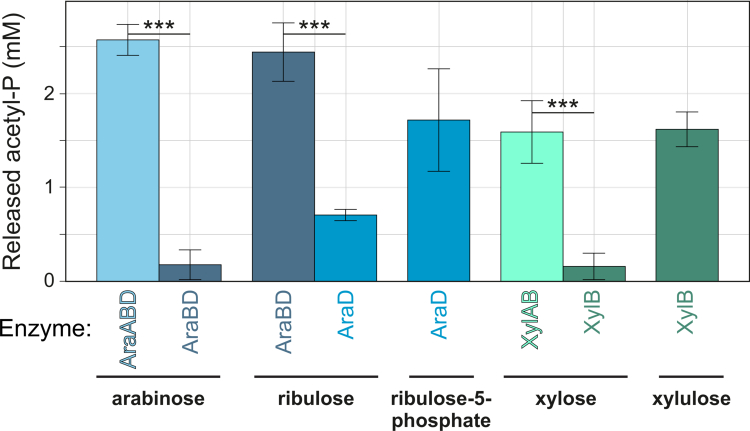
Acetyl-phosphate is released by the activity of AraBDA_*8809*_, XylA_*8809*_, and XylB_*8809*_. Release of acetyl-*P* after 16 h of incubation at 37 °C involving various combinations of the enzymes AraBDA_*8809*His_, XylA_*8809*His_, and XylB_*8809*His,_ all reaction conditions also include XPPKT_*8809*_ for the final conversion to acetyl-phosphate with the corresponding substrates in the metabolic pathway as denoted (*n* = 3). ***: *p* < 0.0005.

The amount of released acetyl-phosphate when AraA_8809His_, AraB_8809His_, and AraD_8809His_ were incubated with 10 mM arabinose as a substrate was significantly higher compared to the same reaction mix in the absence of AraA_8809His_ (*p* < 0.005, Figure 6). Similar results were obtained for ribulose, for which a combination of AraB_8809His_ and AraD_8809His_ resulted in significantly higher production of acetyl-phosphate compared to AraD_8809His_ only (*p* < 0.005). Finally, as expected, a combination of AraD_8809His_ and ribulose-5-phophate led to the release of acetyl-phosphate (Figure 6).

To analyze the *in vitro* activity of XylA_8809_ and XylB_8809_, the corresponding genes were individually cloned into pET28b and purified for subsequent use in hydroxamate assays as described above (Materials and Methods). The amount of released acetyl-phosphate was measured following incubation of various enzyme and substrate combinations in the reaction buffer mix (always including the XPPKT_8809His_ enzyme, which is required for the final conversion of xylulose-5-phosphate into acetyl-phosphate) with either xylose or xylulose. A combination of both enzymes with xylose as a substrate resulted in significantly higher production of acetyl-phosphate compared to only XylB_8809His_ (*p* < 0.005), Figure 6). Concordantly, when supplementing with xylulose as a substrate, only XylB_8809His_ was needed to produce xylulose-5-phosphate, which in turn is used by XPPKT_8809His_ for acetyl-phosphate production (Figure 6). This analysis was not performed for the ribose pathway, because two more enzymes that are part of the bifid shunt would have been required to convert the expected product of RbsK, ribose-phosphate, into xylulose-5-phosphate (Figure 5).

### Phenotypic validation of pentose uptake genes via mutagenesis

**Figure 7.**
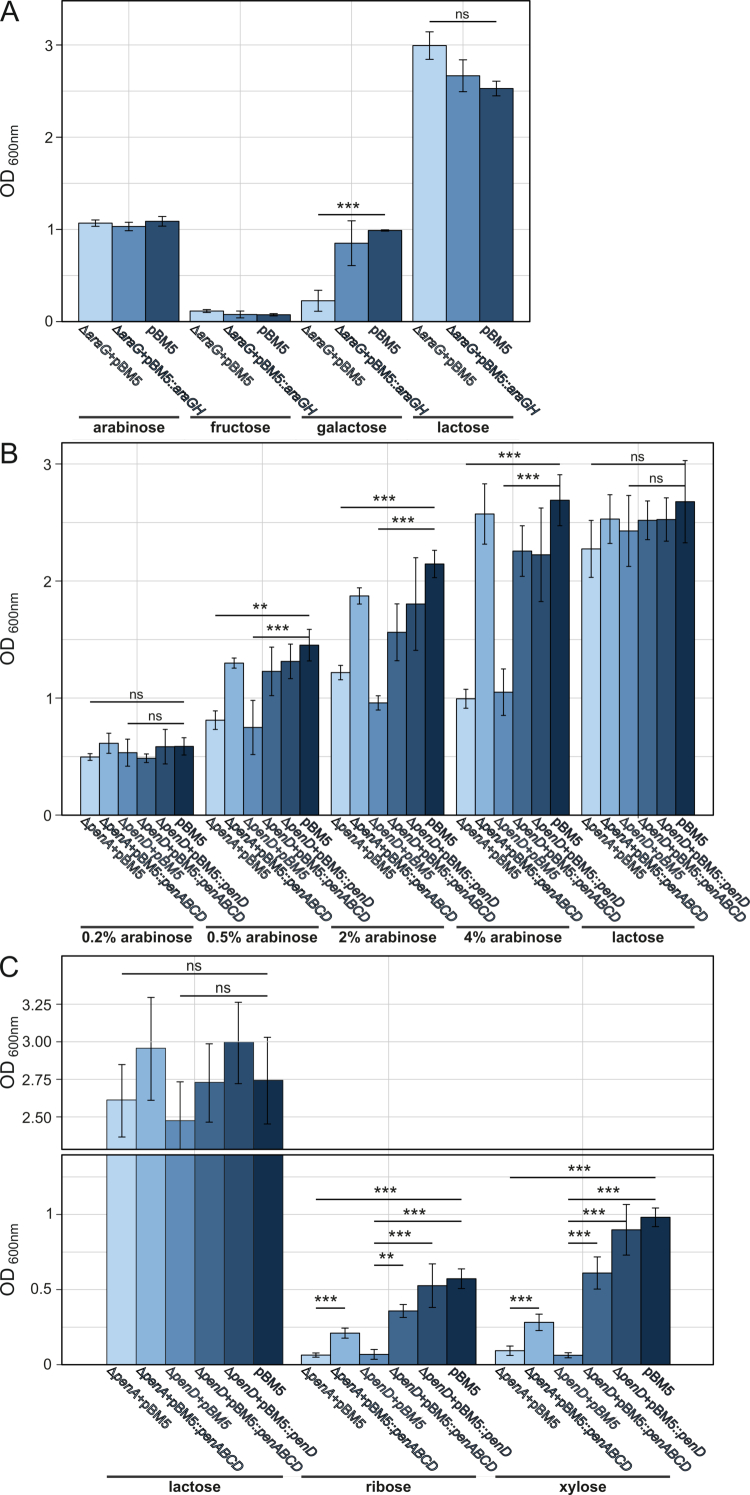
The *araFGH*_*8809*_ and *penABCD* gene clusters are involved in growth in galactose and arabinose/xylose/ribose, respectively. Shown are OD_600 nm_ values after 24 h of anaerobic growth in mMRS supplemented with the carbohydrate sources shown (*n* = 3). (A) *araG*_*8809*_ is required for growth in galactose. Cultures were grown with 0.5% (w/v) lactose, arabinose, fructose, or galactose. (B) *penA*_*8809*_ and *penD*_*8809*_ enhance growth in arabinose at concentrations >0.2% but not in 0.5% (w/v) lactose. (C) *penA*_*8809*_ and *penD*_*8809*_ are required for growth in 0.5% (w/v) xylose and ribose but not lactose. ***: *p* < 0.0005, **: *p* < 0.005, ns: no significance.

To identify pentose-sugar uptake systems of *B. longum* subsp. *longum* NCIMB 8809, we created insertion mutants in genes representing two candidate gene clusters: (i) one that contains homologs of the previously described *araFGH* genes,^26^^,^^50^ and (ii) *penABCD*. While *penABCD* exhibits arabinose/xylose/ribose-dependent upregulation (Figure 2), *araFGH*_*8809*_ was not upregulated in our transcriptomics data during growth in the presence of arabinose (or xylose/ribose). Genetic disruption of the *araG* homolog in *B. longum* subsp. *longum* did not impact growth across a range of arabinose concentrations compared to wild type (Figure 7A). This finding is consistent with a previous study showing that the ROK family repressor AraU in *B. longum* subsp. *longum* NCC2705, which regulates transcription of *araFGH*, is not responsive to arabinose, but rather to fructose and galactose, indicating that this ABC-type transport system is not involved in arabinose uptake.^50^ When *B. longum* subsp. *longum* NCIMB8809-Δ*araG* was cultivated in mMRS medium with galactose as the sole carbohydrate source, growth was significantly lower than wild type (*p* < 0.005), although the mutant still reached a final OD_600 nm_ > 0.4. When genetically complementing this mutant with pBM5:araGH, the resulting strain reaches an OD_600 nm_ similar to that of the wild type when grown in a medium containing galactose. This result suggests that an alternative transporter for galactose is encoded by the genome. The effect on fructose could not be tested since NCIMB 8809 is unable to grow in media containing fructose as the sole carbohydrate source (Figure 7A).

Genetic disruptions of either *penA* or *penD* significantly reduced growth in a medium containing arabinose compared to wild type (*p* < 0.005), but no such effect was observed in a medium containing lactose (Figure 7B). Both mutants were still able to grow to an OD_600 nm_ > 0.4 for certain concentrations of arabinose (Figure 7B). The difference in growth between either mutant and wild type increased depending on the level of arabinose. The difference was not significant in 0.2% arabinose; in contrast, in 4% arabinose, neither mutant was able to grow to an OD_600 nm_ > 1, whereas the wild type strain was shown to reach an OD_600 nm_ > 2 (Figure 7B). Notably, the growth rate of the strains carrying the *penA* or *penD* disruptions was significantly lower than that of the wild type strain (*p* < 0.005) at each assessed arabinose concentration, while there was no effect of the mutations on growth rate in lactose (Supplemental Figure S6). For arabinose concentrations > 0.2%, complementation of either mutant with a plasmid containing the complete *penABCD* gene cluster, or of NCIMB 8809-Δ*penD* with a plasmid containing *penD* restored growth in arabinose similar to that of the wild type strain containing the empty control plasmid pBM5 (Figure 7B).

Our transcriptomic data suggested that *penABCD* is also a good candidate for xylose and ribose uptake, and indeed NCIMB 8809-Δ*penA* and NCIMB 8809-Δ*penD* did not exhibit appreciable growth with xylose or ribose as the sole carbohydrate source (Figure 7C). Introduction of plasmid pBM5:*penD* into NCIMB 8809-Δ*penD* restored growth to that of wild type (Figure 7C). Complementation of NCIMB 8809-Δ*penA* or NCIMB 8809-Δ*penD* with the entire cluster (pBM5:*penABCD*) resulted in a significant increase in OD_600 nm_ (*p* < 0.005); the partial restoration of growth compared to wild type (Figure 7C) can be explained by the plasmid-mediated heterologous expression that may have altered the expression ratio of individual genes of the ABC transporter system. These findings show that *penABCD* is partially responsible for arabinose transport, while also required for xylose and ribose uptake.

### Prevalence of xylose/arabinose/ribose uptake and metabolic genes in members of the *B. longum* subsp. *longum* taxon

tBlastn comparative analysis revealed that all 46 *B. longum* subsp. *longum* strains with publicly available, complete genomes as well as 90 *B. longum* subsp. *longum* strains isolated in the MicrobeMom study harbor the metabolic pathway genes for arabinose and xylose discovered in this study. Furthermore, all strains harbor clear homologs of the *penABCD* cluster (Supplemental Figure S7). All 136 assessed genomes are predicted to encode at least two ribokinases, although only 22 of the 46 strains with publicly available genomes, and 46 of the 90 MicrobeMom strains possess a clear homolog of the B8809_1363–1366 gene cluster upregulated during growth in ribose (Figure 2A,D). Of the 27 strains assayed for growth in ribose (Figure 1), the 13 that were able to grow in ribose as the sole carbohydrate source contain a *rbsK*_*8809*_ homolog, whereas the other 14 strains lacked this gene, consistent with the inability of a ∆*rbsK* mutant to grow in ribose.

## Discussion

Bifidobacteria represent key members of the human gut microbiota and can be found across all stages of host lifespan, having been isolated from nursling stool as well as from centenarians.^51-53^ While a relatively large proportion of their genome is dedicated to the production of glycoside hydrolases and proteins involved in carbohydrate transport or metabolism,^7^ the substrate specificity of many predicted activities has not yet been defined. *B. longum* subsp. *longum* strains are known to utilize various plant-derived glycans, including the two pentose sugars arabinose and xylose.^15-17^ Here, we found that a broad panel of strains can utilize these two carbohydrates, independent of whether they had an adult or infant origin, making it likely that this metabolic capability is a universal and defining trait of the *B. longum* subsp. *longum* taxon. While ribose can be used by a minority of the strains, the presence of a particular ribokinase-encoding gene seems to be key in mediating ribose-dependent growth. Compared to metabolism of hexoses, which enter the bifid shunt at a different point, less ATP is generated for pentose sugar fermentation (theoretical yield is 2.5 moles of ATP versus 2 moles of ATP for 1 mol of fermented hexose or pentose substrate, respectively), and the theoretical ratio of the metabolic end products acetate to lactate also changes (acetate to lactate ratio is 3:2 for hexoses, while being 2:2 for pentoses).^5^^,^^54-56^

Bacteria often prefer glucose over other carbohydrates, but there are known exceptions such as *Streptococcus thermophilus*^57^ and *B. longum* subsp. *longum* NCC2705.^47^ We observed a similar phenomenon in *B. longum s*ubsp*. longum* NCIMB 8809 as that reported for strain NCC2705, in that lactose is fermented in preference of glucose; it was previously found that the glucose transporter is blocked in the presence of lactose, which would explain this preferential carbohydrate utilization.^47^ Furthermore, NCIMB 8809 was shown to co-metabolize arabinose and glucose, as well as arabinose and xylose in which arabinose was initially utilized faster than xylose, potentially because of a secondary uptake system for arabinose. The strain was shown to metabolize glucose at a faster rate than the pentose sugars, and utilized xylose preferentially to ribose, in accordance with its long lag phase in ribose. A possible mechanism for this tiered carbohydrate utilization hierarchy is varying substrate affinities elicited by the solute binding protein of the *penABCD*-encoded ATP-type transporter, although more research is needed to support this idea.

Based on a previous comparative analysis, it had been suggested that an ABC-type transporter system similar to *E. coli* AraFGH is responsible for arabinose uptake in *B. longum* subsp. *longum.*^26^ A knock-out mutation of *araG*_*8809*_ did not affect growth abilities on a medium containing arabinose; however, it significantly reduced growth of galactose, which can be explained by the previously reported recognition of the binding protein of galactose and fructose.^50^ However, our findings clearly show that this similarly named transporter is not involved and that arabinose uptake is instead predominantly modulated by the ABC-type transporter PenABCD, which was previously annotated as a fructose uptake system in *B. longum* subsp. *longum* NCC2705 due to high affinity of the sugar-binding protein with fructose.^58^ We were unable to test this activity in *B. longum* subsp. *longum* NCIMB 8809, because this strain is not able to utilize fructose as the sole carbohydrate source. Two insertion mutants in the *penABCD* gene cluster showed a significant reduction in arabinose-dependent growth, although these mutations did not fully abolish growth on arabinose. The impact of the gene disruptions on the ability to grow in arabinose increased with arabinose concentration, which could be explained by a dual system of arabinose uptake similar to that described for *E. coli*, with one ABC-type transporter system operating at high arabinose concentrations and a second sugar-proton symporter operating at low concentrations.^19^^,^^59^ Such a scenario is consistent with previous observations in *Corynebacterium glutamicum*, in which a mutation in *araE* impacts growth in low arabinose concentrations but not in high concentrations.^60^ Thus, a similar dual system may be in place in *B. longum* subsp. *longum* NCIMB 8809, in which a second transporter imports arabinose when present at low concentration in the environment. This system potentially allows for the strain to discriminate free arabinose and arabinose attached to a more complex glycan that can be found in glycans present in the plant cell wall, such as arabinoxylan, which requires extracellular enzymes for arabinose to be released.^35^ A clear homolog of *E. coli araE,* as a possible alternative arabinose transporter-encoding gene, was not identified in the genome of *B. longum* subsp*. longum.*^26^

Our transcriptomic data demonstrates that the *penABCD*-encoded ABC-type transporter system has affinity for all three pentose sugars as substrates, which is in stark contrast to the situation in *E. coli* in which each substrate is taken up by a separate transport system.^19^^,^^22-24^ We previously showed that this uptake system is upregulated when grown on arabinoxylan, an observation in line with its involvement in the import of extracellularly-cleaved arabinose into the cytoplasm.

In *Bifidobacterium breve* UCC2003, the *rbsACBDK* gene cluster, consisting of an ABC transporter system (*rbsABC*), a ribose mutarotase (*rbsD*), and a ribokinase (*rbsK*), was identified as responsible for the ability to grow on ribose as the sole carbohydrate source.^61^ While a homologous of *rbsK* can be found in *B. longum* subsp. *longum*, this is the only similarity between the two clusters. We have shown that the cluster containing *rbsK*_*8809*_ in *B. longum* subsp. *longum* NCIMB 8809 is required for growth on ribose; however, the true substrate(s) seems to be different as the presence of genes predicted to encode a transporter and a nucleoside hydrolase suggests that this cluster is involved in the utilization of nucleotides/nucleosides. Similar genes to the *rbsK* cluster can be found in *E. coli*, in which RihC catalyzes the hydrolysis of both purine and pyrimidine ribonucleosides to produce ribose and the corresponding base. The presence of this cluster suggests the ability of certain *B. longum* subsp. *longum* strains to utilize nucleosides for growth, although *B. longum* subsp. *longum* NCIMB 8809, *B. longum* subsp. *longum* JCM1217, and seven MicrobeMom strains harboring this cluster were unable to grow in a medium containing cytidine, inosine, or uridine as the sole carbohydrate source (Supplemental Figure S3).

While many bifidobacterial strains are typically isolated from either adult or infant feces depending on carbohydrate preference, *B. longum* subsp. *longum* strains are routinely isolated from both babies and adults.^62^ They typically are able to utilize lacto-N-tetraose as the sole carbohydrate source, which is a common human milk-oligosaccharide and can be found in the diet of breastfed infants.^63^ While arabinose and xylose do not represent carbohydrates that are part of the infant diet, we have shown here that the ability to utilize them is independent of the strain origin (i.e., infant or adult). Therefore, during the weaning period *B. longum* subsp. *longum* is able to switch from HMOs and its derived carbohydrates to plant-based glycans and persist in this changed gut environment. This ability may explain why this bifidobacterial species is so prevalent across all host age groups.^51-53^ Furthermore, the identified hierarchy and metabolic flexibility exhibited by *B. longum* might be a response to address competitive interactions with other gut microbes. A preferential use of hexose sugars over pentose sugars may be due to a higher energy yield of the former compared to pentose sugars. While the use of lactose over glucose may be explained by their expected presence in the human gut: lactose is the most abundant solid component in human breast milk, while it is also a constituent part of all human milk oligosaccharides, and is therefore expected to be available at high levels for bifidobacteria. In contrast, glucose, if present as a monosaccharide in the diet, will be quickly absorbed by the host, or will only become available following degradation of larger polysaccharides. Similarly, pentose sugars may be present in the gut environment at varying levels and this may have guided their preferential utilization by particular bifidobacterial species. Co-metabolism of glucose and arabinose can also help outcompete other bacteria, often repressing metabolic genes unrelated to glucose metabolism in the presence of glucose, by utilizing different substrates at the same time. Furthermore, *B. longum* NCIMB 8809 exhibits a higher growth rate for lactose compared to arabinose and the other pentose sugars, perhaps because of the higher energy yield of lactose, in turn allowing for higher competitiveness. Our study clearly highlights the need for experimental validation of sequence-based predictions, as *in silico* comparative predictions are not always accurate. This need motivates the development of advanced molecular and genetic tools to precisely determine or indeed validate the function of metabolic genes, such as the transposon mutant libraries recently generated for *B. longum* subsp. *longum*^38^ and *Bifidobacterium breve.*^64^ Furthermore, the ability to utilize arabinose and xylose could be exploited to develop prebiotics to specifically enrich *B. longum* subsp. *longum*, similar to a recent finding that an arabinoxylan-rich diet increased the relative abundance of *B. longum* subsp. *longum.*^65^^,^^66^ Nonetheless, it is important to be cognizant of the possibility that certain metabolic capabilities may vary across *B. longum* subsp. *longum* strains. Such variation was shown here for ribose metabolism, and has also been shown for larger, complex plant-derived glycans such as arabinoxylan and arabinan.^35^ Therefore, it is important to take these strain-specific traits into account when developing a synbiotic so as to activate beneficial activities at the strain level.

## Supplementary Material

KGMI-S-2025-2467.R1 (258834176.R1) - Supplementary.docx KGMI-S-2025-2467.R1 (258834176.R1) - Supplementary.docx

## Data Availability

The sequencing data that support the findings of this study are available at https://www.ncbi.nlm.nih.gov/ within BioProject PRJNA1082215, under SRR35316394-SRR35316398.
