# Supplementary material for: Metabolic pathway analysis reveals hierarchical pentose sugar utilization and metabolic flexibility of Bifidobacterium longum
Source: Gut Microbes. 2026 Mar 23;18(1):2647591. doi: 10.1080/19490976.2026.2647591 (PMC13011590; doi:10.1080/19490976.2026.2647591)
Supplement: KGMI-S-2025-2467.R1 (258834176.R1) - Supplementary.docx [file KGMI_A_2647591_SM9375.docx]

**Supplementary Information for** **“Metabolic pathway analysis and hierarchical order of pentose sugar utilization by *Bifidobacterium longum* subsp. *longum* NCIMB 8809”**

**Authors:** Lisa Friess^1^, Fionnuala M. McAuliffe^2^, Paul D. Cotter^1,3^, Anthony L. Shiver^4^, Kerwyn Casey Huang^4,5,6^, Anne de Jong^7^, and Douwe van Sinderen^1*^

**Affiliations:**

^1^APC Microbiome Ireland & School of Microbiology, University College Cork, Western Road, Ireland

^2^UCD Perinatal Research Centre, School of Medicine, University College Dublin, National Maternity Hospital, Dublin, Ireland

^3^Teagasc Food Research Centre, Cork, Ireland

^4^Department of Bioengineering, Stanford University, Stanford, USA

^5^Department of Microbiology and Immunology, Stanford University School of Medicine, Stanford, USA

^6^Chan-Zuckerberg Biohub, San Francisco, USA

^7^Department of Molecular Genetics, University of Groningen, Groningen, the Netherlands

*Corresponding author: [d.vansinderen@ucc.ie](mailto:d.vansinderen@ucc.ie)

**Supplementary Tables**

# Supplementary Table 1: Bacterial strains and plasmids used in this study.

| **Strain** | **Features** | **Refences/source** |
| --- | --- | --- |
| ***B. longum* subsp*. longum*** |  |  |
| NCIMB 8809 | isolated from baby stool | NCIMB/ CP011964.1 |
| JCM 1217 | Isolated from adult stool | JCM/ CP072501.1 |
| MB0044 | isolated from baby stool | CP146844 ^1^ |
| MB0212 | isolated from baby stool | CP157377-CP157378 ^1^ |
| MB0308 | isolated from baby stool | CP146700 ^1^ |
| MB0318 | isolated from baby stool | CP146699 ^1^ |
| MM0003 | isolated from adult stool | CP146904-CP146907 ^1^ |
| MM0024 | isolated from adult stool | CP146639-CP146641 ^1^ |
| MM0160 | isolated from adult stool | CP146698 ^1^ |
| MM0259 | isolated from adult stool | CP155475 ^1^ |
| MM0286 | isolated from adult stool | CP146642-CP146644 ^1^ |
| MM0289 | isolated from adult stool | CP146901-CP146903 ^1^ |
| MM0302 | isolated from adult stool | CP146696 ^1^ |
| MM0307 | isolated from adult stool | CP146695 ^1^ |
| MM0321 | isolated from adult stool | CP146636-CP146638 ^1^ |
| MM0360 | isolated from adult stool | CP146634-CP146635 ^1^ |
| MM0362 | isolated from adult stool | CP146630-CP146633 ^1^ |
| MM0364 | isolated from adult stool | CP146694 ^1^ |
| MM0369 | isolated from adult stool | CP146491-CP146492 ^1^ |
| MM0375 | isolated from adult stool | CP146647-CP146649 ^1^ |
| MM0380 | isolated from adult stool | CP146484 ^1^ |
| MM0441 | isolated from adult stool | CP146496-CP146497 ^1^ |
| MM0450 | isolated from adult stool | CP146493-CP146495^1^ |
| MM0464 | isolated from adult stool | CP146483 ^1^ |
| MM0465 | isolated from adult stool | CP146485 ^1^ |
| MM0492 | isolated from adult stool | CP146645-CP146646 ^1^ |
| MM0494 | isolated from adult stool | CP146487-CP146490 ^1^ |
| NCIMB 8809 + pBM5 | NCIMB 8809 containing pBM5 | this work |
| NCIMB 8809 ∆*araD* + pBM5 | NCIMB 8809 with an insertion in *araD* containing pBM5 | this work |
| NCIMB 8809 Δ*araD* + pBM5::*araD* | NCIMB 8809 with an insertion in *araD* containing pBM5::*araD* | this work |
| NCIMB 8809 Δ*araD* + pBM5::*araDA* | NCIMB 8809 with an insertion in *araD* containing pBM5::*araDA* | this work |
| NCIMB 8809 Δ*araA* + pBM5 | NCIMB 8809 with an insertion in *araA* containing pBM5 | this work |
| NCIMB 8809 Δ*araA* + pBM5::*araA* | NCIMB 8809 with an insertion in *araA* containing pBM5::*araA* | this work |
| NCIMB 8809 Δ*araG* + pBM5 | NCIMB 8809 with an insertion in *araG* containing pBM5 | this work |
| NCIMB 8809 Δ*araA* + pBM5::*araGH* | NCIMB 8809 with an insertion in *araG* and containing plasmid pBM5::*araGH* | this work |
| NCIMB 8809 Δ*xylA* + pBM5 | NCIMB 8809 with an insertion in *xylA* containing pBM5 | this work |
| NCIMB 8809 Δ*xylA* + pBM5::*xylA* | NCIMB 8809 with an insertion in *xylA* containing pBM5::*xylA* | this work |
| NCIMB 8809 Δ*xylB* + pBM5 | NCIMB 8809 with an insertion in *xylB* containing pBM5 | this work |
| NCIMB 8809 Δ*xylB* + pBM5::*xylB* | NCIMB 8809 with an insertion in *xylB* containing pBM5::*xylB* | this work |
| NCIMB 8809 Δ*rbsK* + pBM5 | NCIMB 8809 with an insertion in *rbsK* containing pBM5 | this work |
| NCIMB 8809 Δ*rbsK* + pBM5::*rbsK* | NCIMB 8809 with an insertion in *rbsK* containing pBM5::*rbsK* | this work |
| NCIMB 8809 Δ*penA* + pBM5 | NCIMB 8809 with an insertion in *penA* containing pBM5 | this work |
| NCIMB 8809 Δ*penA* + pBM5::*penABCD* | NCIMB 8809 with an insertion in *penA* containing pBM5::*penABCD* | this work |
| NCIMB 8809 Δ*penD* + pBM5 | NCIMB 8809 with an insertion in *penD* containing pBM5 | this work |
| NCIMB 8809 Δ*penD* + pBM5::*penD* | NCIMB 8809 with an insertion in *penD* containing pBM5::*penD* | this work |
| NCIMB 8809 Δ*penD* + pBM5::*penABCD* | NCIMB 8809 with an insertion in *penD* containing pBM5::*penABCD* | this work |
| ***E. coli*** |  |  |
| EC101 | cloning host, *repA*^+^ *km^r^* | ^2^ |
| DH5α | cloning host, *recA1* | Invitrogen |
| BL21 | cloning host, T7^-^ | Stratagene |
| **Plasmids** |  |  |
| pET28b | *E.coli* expression vector with N-terminal His tag | Novagen |
| pET28b::*araA* | pET28b harbouring *araA* | this work |
| pET28b::*araB* | pET28b harbouring *araB* | this work |
| pET28b::*araD* | pET28b harbouring *araD* | this work |
| pET28b::*xylA* | pET28b harbouring *xylA* | this work |
| pET28b::*xylB* | pET28b harbouring *xylA* | this work |
| pET28b:*xppkt* | pET28b harbouring *xppkt* | this work |
| pFREM2 | pFREM28, R-M motif free for *B. longum* | ^3^ |
| pFREM2::*araA* | pFREM2 harbouring an internal fragment of *araA* | this work |
| pFREM2::*araD* | pFREM2 harbouring an internal fragment of *araD* | this work |
| pFREM2::*xylA* | pFREM2 harbouring an internal fragment of *xylA* | this work |
| pFREM2::*xylB* | pFREM2 harbouring an internal fragment of *xylB* | this work |
| pFREM2::*rbsK* | pFREM2 harbouring an internal fragment of *rbsK* | this work |
| pFREM2::*penA* | pFREM2 harbouring an internal fragment of *penA* | this work |
| pFREM2:: *penD* | pFREM2 harbouring an internal fragment of *penB* | this work |
| pFREM2:: *araG* | pFREM2 harbouring an internal fragment of *araG* | this work |
| pBM5 | pBC1, pUC19-Tc^r^ | ^3^ |
| pBM5::*araA* | pBM5 harbouring *araA* + p44 promoter | this work |
| pBM5::*araD* | pBM5 harbouring *araD* + p44 promoter | this work |
| pBM5::*araDA* | pBM5::*araD* and *araA* + p44 promoter | this work |
| pBM5::*xylA* | pBM5 harbouring *xylA* | this work |
| pBM5::*xylB* | pBM5 harbouring *xylB* | this work |
| pBM5::*rbsK* | pBM5 harbouring *rbsK* + p44 promoter | this work |
| pBM5::*penABCD* | pBM5 harbouring *penABCD* | this work |
| pBM5::*penD* | pBM5 harbouring *penD* + p44 promoter | this work |
| pBM5::*araGH* | pBM5 harbouring *araGH* + p44 promoter | this work |
| pNZEM | Gene expression vector, Em^r^ | ^4^ |

# Supplementary Table 2: Factor table and comparison used for transcriptomic analyis.

| **Sample** | **Factor** |
| --- | --- |
| Sample 1 | NCIMB 8809 –Lac-1 |
| Sample 2 | NCIMB 8809 –Lac-1 |
| Sample 3 | NCIMB 8809 -Ara |
| Sample 4 | NCIMB 8809 -Ara |
| Sample 5 | NCIMB 8809 -Rib |
| Sample 6 | NCIMB 8809 -Rib |
| Sample 7 | NCIMB 8809 –Lac-2 |
| Sample 8 | NCIMB 8809 –Lac-2 |
| Sample 9 | NCIMB 8809 –Xyl |
| Sample 10 | NCIMB 8809 –Xyl |
| **Comparisons** | |
| NCIMB 8809 -Ara | NCIMB 8809 –Lac-1 |
| NCIMB 8809 -Rib | NCIMB 8809 –Lac-1 |
| NCIMB 8809 –Xyl | NCIMB 8809 –Lac-2 |

# Supplementary Table 3: Information about genes used to generate and confirm insertion mutants.

| **Gene** | **Locus tag** | **Length of insert** | **Codons** | **Location in genome** | **Primers** | **Tm** |
| --- | --- | --- | --- | --- | --- | --- |
| *araD* | B8809_0307 | 347 bp | 62 to 177  (of 259) | 404,001 to 404,348 | gtcgtgtggaccgcgggcaa  gcctcggagccgatcagacgg | 72 ℃ |
|  |  | 676 kb |  | Before B8809_307  pFREM2 | ctttctgacctccctctattgagggagg  gccaacgttttcgccaacg | 55 ℃ |
| *araA* | B8809_0308 | 409 bp | 28 to 165 (of 506) | 404,910 to 405,317 | ggtcgccattcactccgctg  ggtgccgagcttctcggc | 72 ℃ |
|  |  | 734 bp |  | Before B8809_308  pFREM2 | ggagctgtcagcgatagctgac  gccaacgttttcgccaacg | 55 ℃ |
| *penA* | B8809_1288 | 538 bp | 8 to 187 (of 328) | 1,610,624 to 1,611,162 | gccctcgttgcttctgctgc  cccaaccagtgccacgatcc | 72 ℃ |
|  |  | 786 bp |  | Before B8809_1288  pFREM2 | gaggctgtcgatgacgaaca gccaacgttttcgccaacg | 54 ℃ |
| *penD* | B8809_1285 | 410 bp | 5 to 141 (of 341) | 1,607,029 to 1,607,438 | cggcaaacaaagtgaaggctcc  gaacatcgtcgacaaggtcgc | 70 ℃ |
|  |  | 893 bp |  | B8809_1285  pFREM2 | cttgaccggcagacccatg  ttgctatcgtcccgtctcctataac | 54 ℃ |
| *rbsK* | B8809_1364 | 454 bp | 27 to 178 (of 323) | 1,706,828 to 1,707,282 | cgaatggcgaatcgttgagca  ggtcgttactgagcggttgc | 69 ℃ |
|  |  | 1.5 kb |  | B8809_1363  pFREM2 | cgataattatcgggcaggc  ttgctatcgtcccgtctcctataac | 50 ℃ |
| *xylB* | B8809_1427 | 515 bp | 91 to 263 (of 507) | 1,790,654 to 1,791,155 | ggaatgacaccagttccgc  gccttcaacgtccttgcc | 67 ℃ |
|  |  | 1.1 kb |  | B8809_1427  pFREM2 | cgctcacatccgtctatcac  gccaacgttttcgccaacg | 52 ℃ |
| *xylA* | B8809_1440 | 457 bp | 27 to 179 (of 450) | 1,806,868 to 1,807,324 | gatgccgacaaggtcgttgc  ggcgcttgccgatctccaag | 71 ℃ |
|  |  | 1.2 kb |  | B8809_1441  pFREM2 | cgtcgaactcgtgcatcaac  gccaacgttttcgccaacg | 53 ℃ |
| *araG* | B8809_1449 | 441 bp | 32 to 179 (of 518) | 1,814,184 to 1,814,624 | gcgagatccacgccatctgc  cctcatcgttcagtgcagcg | 70 ℃ |
|  |  | 894 bp |  | B8809_1450  pFREM2 | gacgtcccgtccaagctg  ttgctatcgtcccgtctcctataac | 54 ℃ |

# Supplementary Table 4: Information about complementation of insertion mutations.

| **Gene** | **Locus_tag** | **Artificial promoter?** | **Length** | **Location in genome** | **Primers** | **Tm** |
| --- | --- | --- | --- | --- | --- | --- |
| *araD* | B8809_0307 | yes | 705 bp | 403,893 to 404,598 | ccaataatggcaactttggc  catcaaatcagtgctgaccg | 63 ℃ |
| *araA* | B8809_0308 | yes | 1.5 kB | 404,817 to 406,345 | aaccctatggttatggaaaatcc  gccaatcagtgctggttg | 63 ℃ |
| *araDA* | B8809_0307 & B8809_0308 | yes | 2.5 kB | 403,893 to 406,345 | ccaataatggcaactttggc  gccaatcagtgctggttg | 63 ℃ |
| *penABCD* | B8809_1285 - B8809_1288 | no | 5 kB | 1,606,355 to 1,611,362 | caaggcaaaacagtgttgc  gtgaccttgccatcacag | 64 ℃ |
| *penD* | B8809_1288 | yes | 1 kB | 1,607,462 to 1,606,425 | ggcggtggcagcatg  cccgctattttttatctccgc | 65 ℃ |
| *rbsK* | B8809_1364 | yes | 979 bp | 1,706,390 to 1,707,369 | atgcatcatgtcagcatcatg  gtaatcaattcgtaccaaaccg | 62 ℃ |
| *xylB* | B8809_1427 | no | 1.7 kB | 1,790,205 to 1,791,912 | Cgctcacatccgtctatcac  gctggcagcgtatcagc | 66 ℃ |
| *xylA* | B8809_1440 | no | 1.6 kB | 1,806,050 to 1,807,630 | cgaggaattgcgtgacgtc  cgatggtcactcgacctcag | 67 ℃ |
| *araGH* | B8809_1449 & B8809_1450 | yes | 2.8 kB | 1,811,946 to 1,814,727 | ggtgacatgtcatcaaatgac  gaaggtcagctcttcttgc | 62 ℃ |

# Supplementary Table 5: Information about genes cloned into pET28b for protein purification.

| **Gene** | **Locus tag** | **Length** | **Location in genome** | **Primers** | **Tm** |
| --- | --- | --- | --- | --- | --- |
| *araB* | B8809_0306 | 1.7 kb | 402,168 to 403,814 | atggcagcaaccgacaac  ggcttacttatcttcgaggttgatg | 66 ℃ |
| *araD* | B8809_0307 | 707 bp | 403,899 to 404,605 | atggcaactttggctgattac  gacactccatcaaatcagtgc | 64 ℃ |
| *araA* | B8809_0308 | 1.5 kb | 404,823 to 406,334 | atggttatggaaaatcccttcgag  gctccgccaatcagtgc | 65 ℃ |
| *xylB* | B8809_1427 | 1.5 kb | 1,790,380 to 1,791,912 | atgacgagagtactggttgc  gctggcagcgtatcagc | 65 ℃ |
| *xylA* | B8809_1440 | 1.4 kb | 1,806,050 to 1,807,405 | atgggtctgtgggatgttgac  cgatggtcactcgacctcag | 68 ℃ |
| *XPPKT* | B8809_0636 | 2.5 kb | 829,356 to 828,824 | atgacgagtcctgttattggc  tggaggtgttatcactcgttg | 65 ℃ |

# Supplementary Table 6: Comparative analysis of metabolic genes.

| **Gene in *B. longum* subsp. *longum* NCIMB 8809** | **Compared to** | **Amino acid similarity** | **Significance (e-value)** | **Function** |
| --- | --- | --- | --- | --- |
| *araA_8809_* | *E. coli* | 45.05% | 7e-162 | converts Ara to ribulose |
| *araB_8809_* | *E. coli* | 29.51% | 2e-04 | converts ribulose to ribulose-5-phosphate |
| *araD_8809_* | *E. coli* | 41.38% | 5e-43 | converts ribulose-5-phosphate to xylulose-5-phosphate |
| *xylA_8809_* | *E. coli* | 43.28% | 1e-136 | converts Xyl to xylulose |
| *xylB_8809_* | *E. coli* | 30.06% | 2e-44 | converts xylulose to xylulose-5-phosphate |
| *rbsK_8809_* | *Bifidobacterium breve* | 93.50% | 0.0 | converts Rib to ribose-5-phosphate |
| *dcuC_8809_* | *dcuC* in *E. coli* | 34.17% | 3e-72 | efflux carrier that exports C₄-dicarboxylic acids |
| *rihC*_8809_ | *rihC* in *E. coli* | 47.84% | 9e-95 | cleaves ribonucleosides into Rib and nitrogenous bases |

# Supplementary Figures


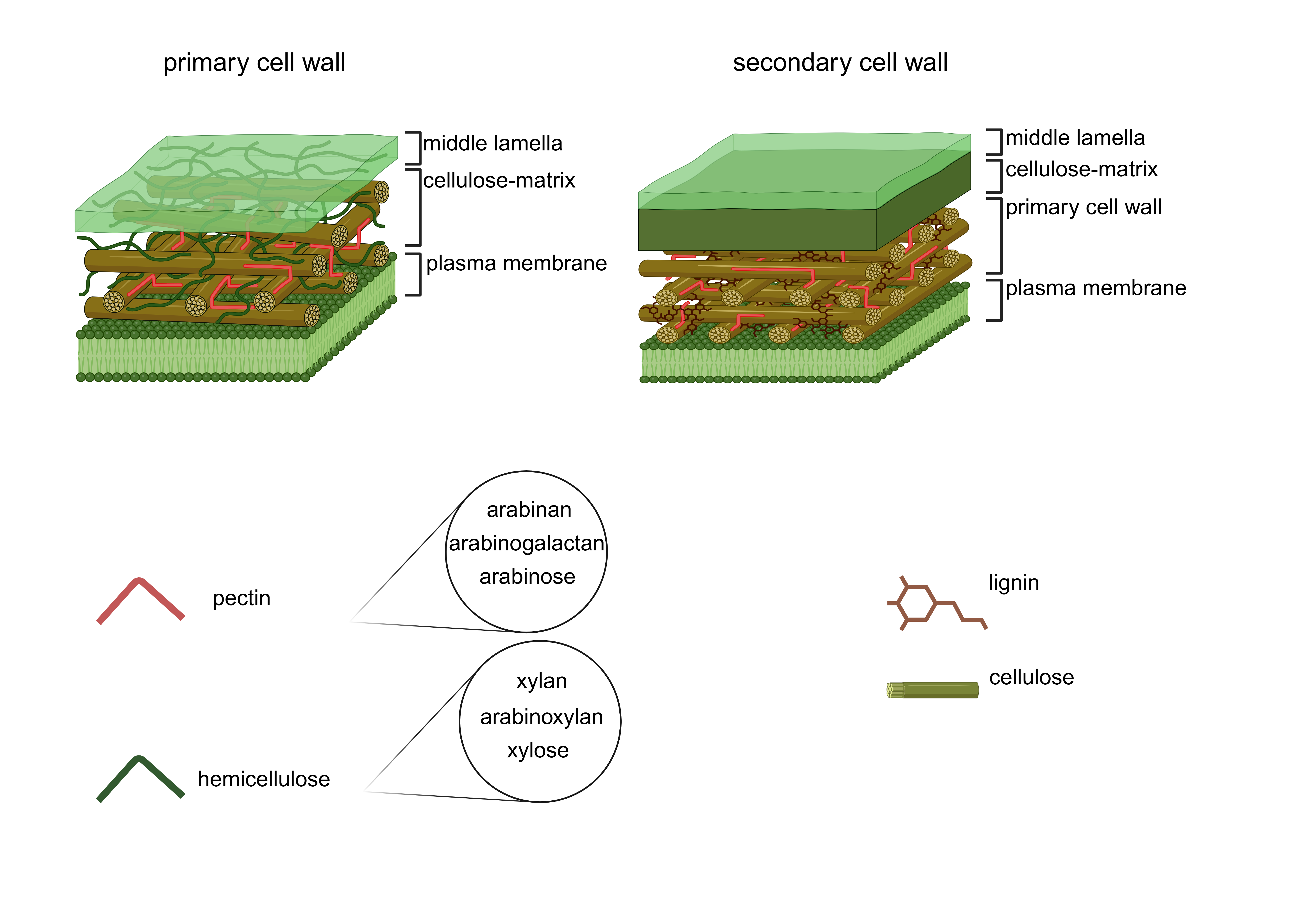


# Figure S1: Schematic of the structure of a plant cell wall^5^. Created in BioRender.

**
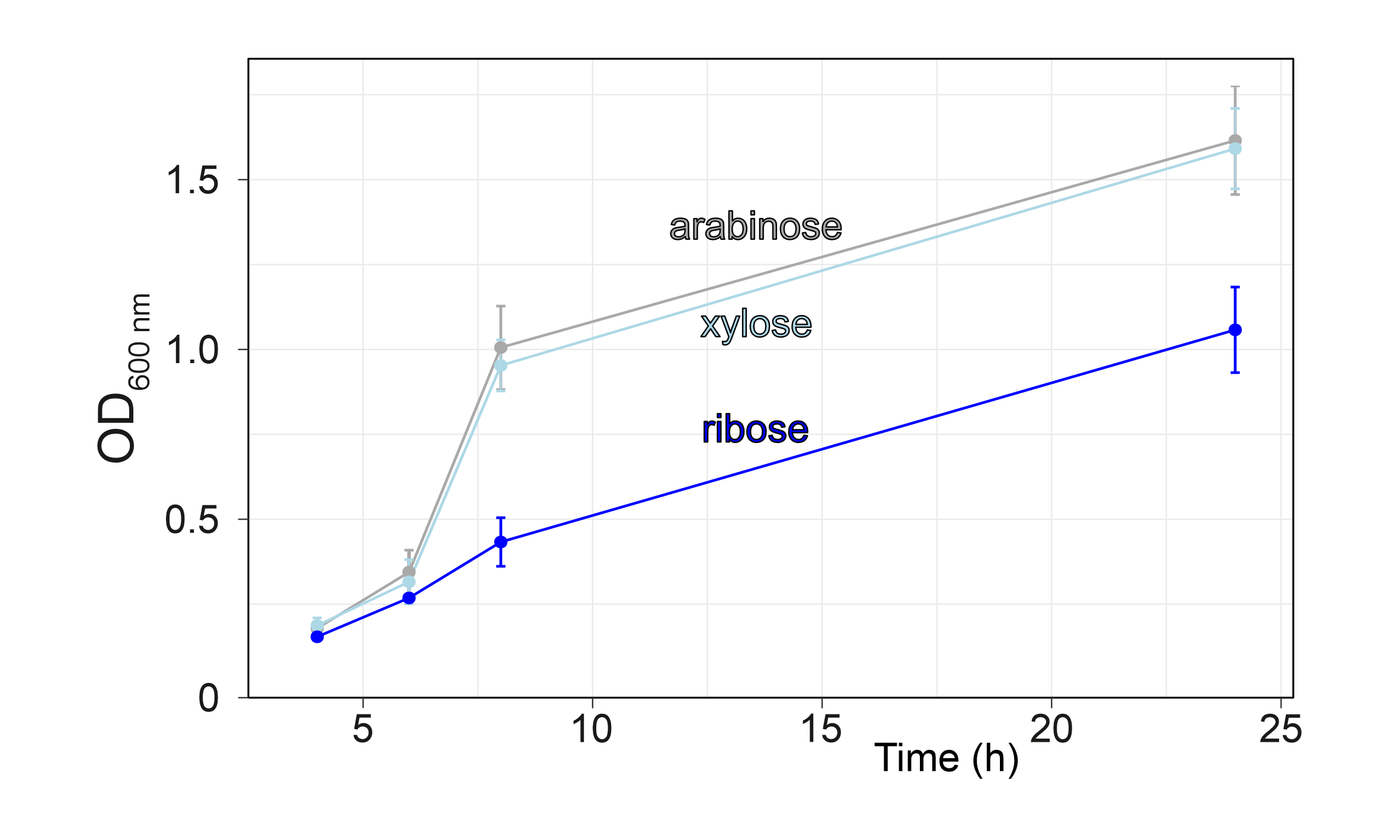
**

**Figure S2: Growth curve of *B. longum* subsp. *longum* NCIMB 8809 during cultivation with arabinose, xylose and ribose.**

#
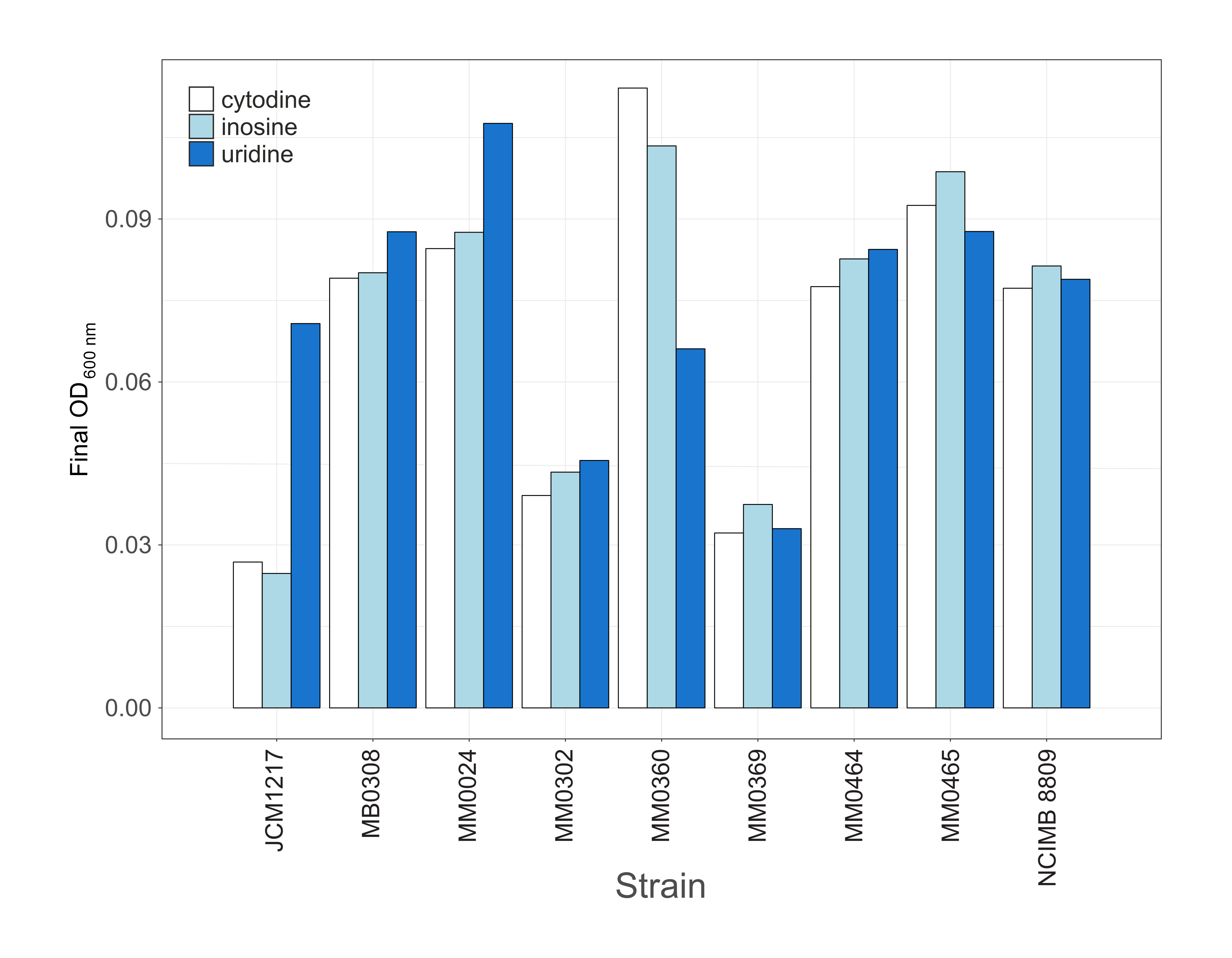
Figure S2: Growth yield of *B. longum* subsp. *longum* strains during cultivation with various nucleosides.


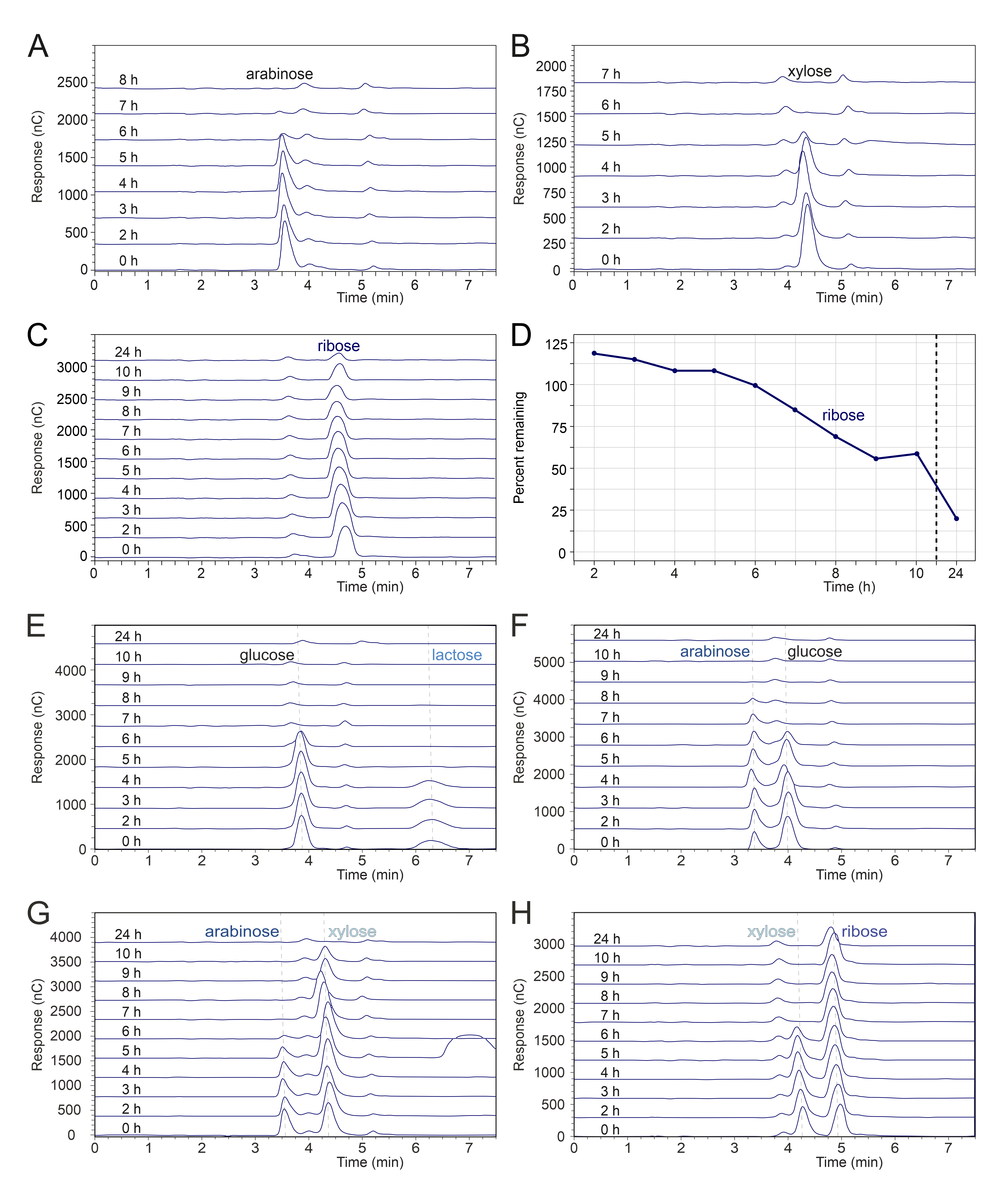


# Figure S3: HPAEC-PAD analysis of the supernatant of *B. longum* subsp*. longum* NCIMB 8809.


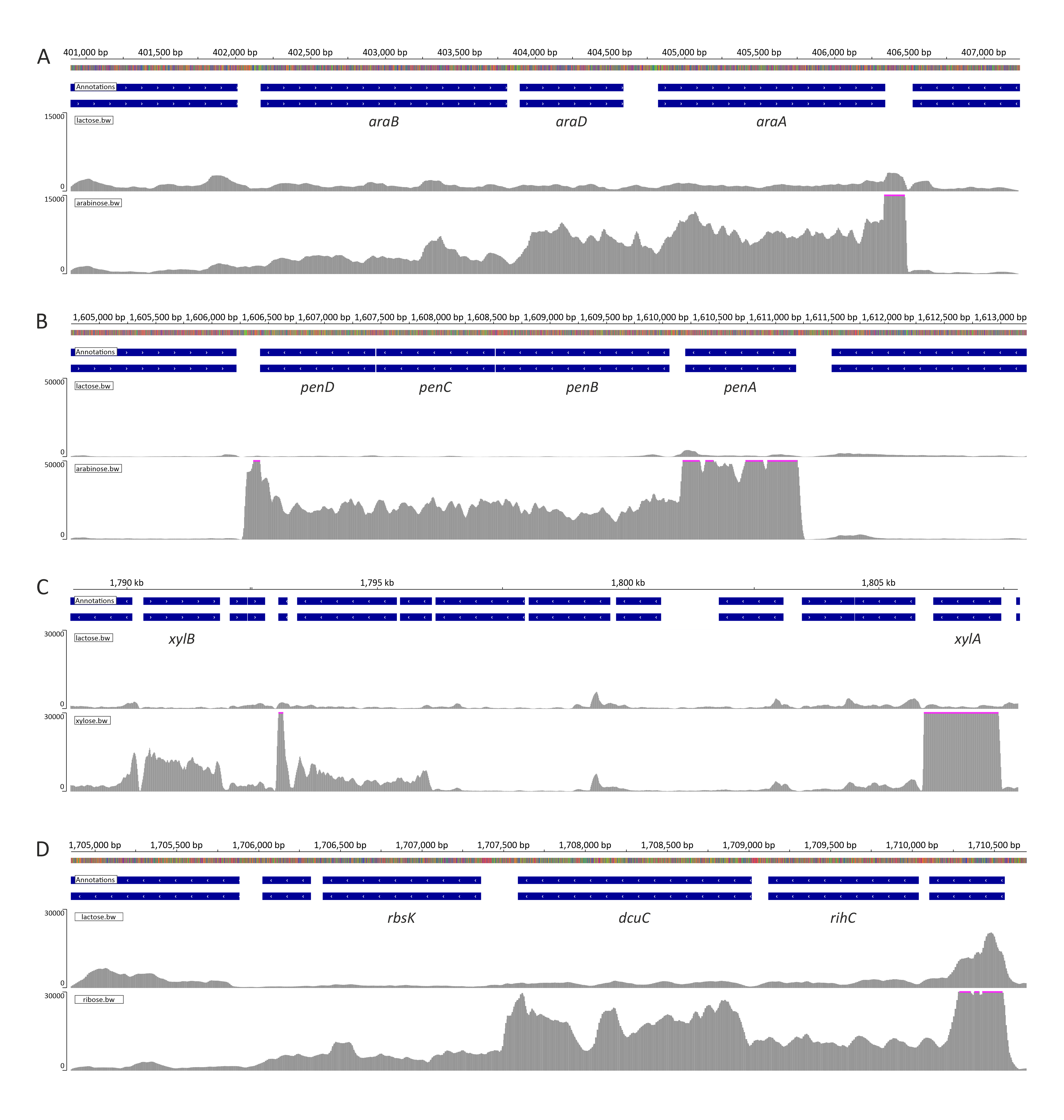


**Figure S4: Gene clusters involved in utilisation of arabinose, xylose and ribose**.

**
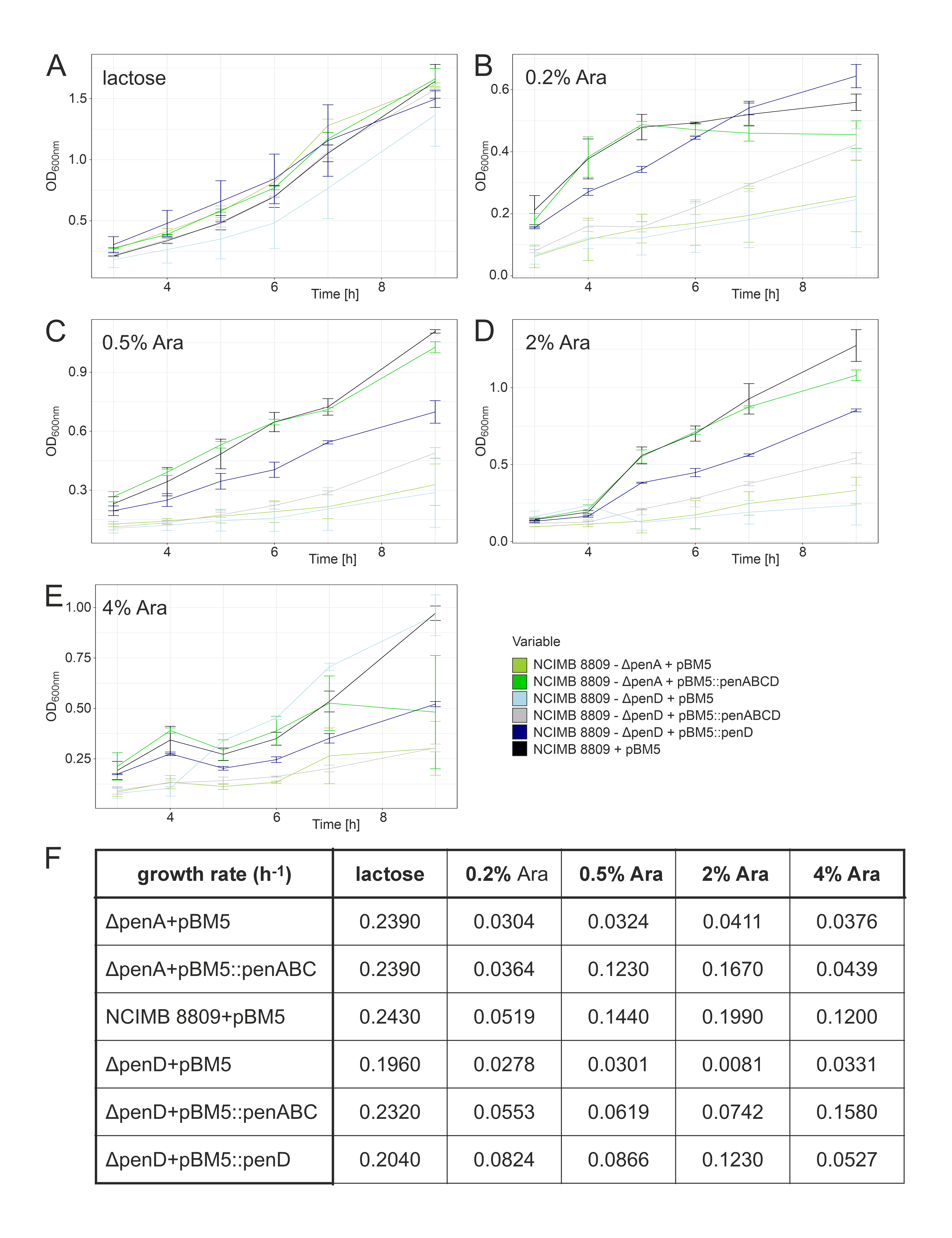
Figure S5: Growth dynamics of *B. longum* subsp. *longum* NCIMB 8809 strains across media.**

## **
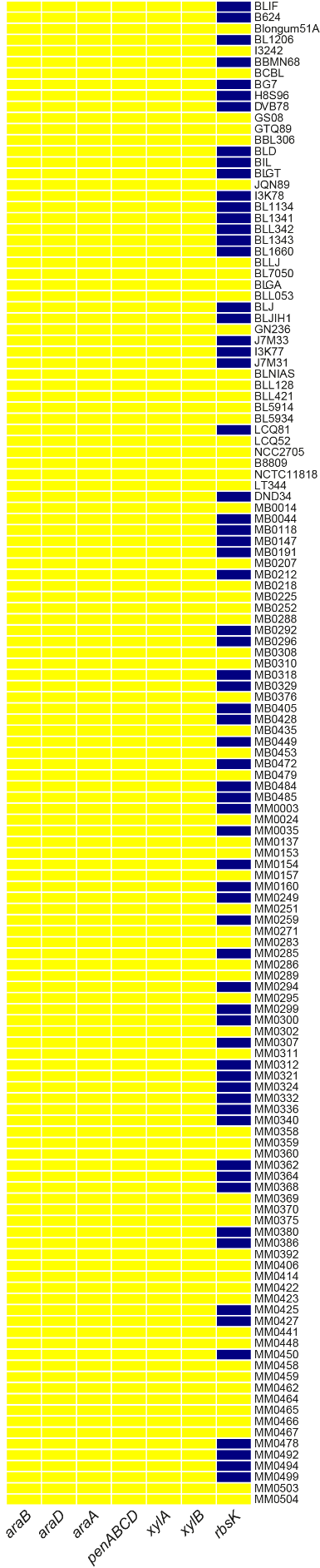
**

# Figure S6: Presence (yellow) or absence (blue) of genes required for growth on arabinose, xylose, and ribose across *B. longum* subsp. *longum* strains.

# Supplementary Figure Legends

**Figure S1: Schematic of the structure of a plant cell wall**^5^. Created in BioRender.

**Figure S2: Growth curve of B. longum subsp. longum NCIMB 8809 in mMRS supplemented with arabinose, xylose and ribose**. Cultures were grown for 24 hours OD_600 nm_ values were measured using a spectrophotometer on culture samples that were taken after 4, 6, 8 and 24 hours of growth (n=3).

**Figure S3: Growth yield of *B. longum* subsp. *longum* strains during cultivation with various nucleosides.** Cultures were grown for 24 h in mMRS with the indicated nucleoside. Final OD_600 nm_ was measured in a spectrophotometer (n=2).

**Figure S4: HPAEC-PAD analysis of the supernatant of *B. longum* subsp*. longum* NCIMB 8809.** (A-C) Cultures were grown for 24 h in mMRS containing 0.25% (w/v) arabinose (A), 0.25% (w/v) xylose (B), or 0.25% (w/v) ribose (C) (n=4). Samples were taken over time as indicated on each panel. (D) Remaining levels of ribose for the cultures in (C). (E-H) Cultures were grown for 24 h in mMRS containing mixed carbohydrates: 0.25% (w/v) lactose + 0.25% (w/v) glucose (E), 0.25% (w/v) glucose + 0.25% (w/v) arabinose (F), 0.25% (w/v) arabinose + 0.25% (w/v) xylose (G), and 0.25% (w/v) xylose + 0.25% (w/v) ribose (H) (n=4). Samples were taken over time as indicated on each panel.

**Figure S5: Gene clusters involved in utilisation of arabinose, xylose and ribose**. RNA-seq data were visualized using igv.org/app/. (A) *araBDA* cluster for growth on lactose and arabinose. (B) *penABCD* cluster for growth on lactose and arabinose. (C) Gene cluster containing *xylA* and xylB for growth on lactose and xylose. (D) Gene cluster containing *rbsK* for growth on lactose and ribose.

**Figure S6: Growth dynamics of *B. longum* subsp. *longum* NCIMB 8809 strains across media.** (A-E) Cultures were grown for 10 h in mMRS containing 0.5% (w/v) lactose (A), 0.2% (w/v) arabinose (B), 0.5% (w/v) arabinose (C), 2% (w/v) arabinose (D), or 4% (w/v) arabinose (E) (n=3). Optical density (OD) at 600 nm was measured every hour. (F) Maximum growth rates for the growth curves in (A-E) (Methods).

**Figure S7: Presence (yellow) or absence (blue) of genes required for growth on arabinose, xylose, and ribose across *B. longum* subsp. *longum* strains.** Homologues were identified using BlastX analysis^6^ for 90 *B. longum* subsp. *longum* strains isolated in the MicrobeMom study together with 46 publicly available strains on NCBI (using a 80% aa identify over 95% coverage cut off, E-value: 1e-20). Only *rbsK* is not fully conserved.

# References

1. Feehily C, O’Neill IJ, Walsh CJ, Moore RL, Killeen SL, Geraghty AA, Lawton EM, Byrne D, Sanchez-Gallardo R, Nori SRC, et al. Detailed mapping of *Bifidobacterium* strain transmission from mother to infant via a dual culture-based and metagenomic approach. *Nat Commun*. 2023;14(1). doi:10.1038/s41467-023-38694-0

2. Law J, Buist G, Haandrikman A, Kok J, Venema G, Leenhouts K. A. A system to generate chromosomal mutations in *Lactococcus lactis* which allows fast analysis of targeted genes. *J Bacteriol*. 1995;177(24):7011-7018. doi:10.1128/jb.177.24.7011-7018.1995

3. Friess L, Bottacini F, McAuliffe FM, O’Neill IJ, Cotter PD, Lee C, Munoz-Munoz J, van Sinderen D. Two extracellular α-arabinofuranosidases are required for cereal-derived arabinoxylan metabolism by *Bifidobacterium longum* subsp. *longum*. *Gut Microbes*. 2024;16(1):2353229. doi:10.1080/19490976.2024.2353229

4. Hoedt EC, Bottacini F, Cash N, Bongers RS, van Limpt K, Ben Amor K, Knol J, MacSharry J, van Sinderen D. Broad Purpose Vector for Site-Directed Insertional Mutagenesis in *Bifidobacterium breve*. *Front Microbiol*. 2021;12. doi:10.3389/fmicb.2021.636822

5. Loix C, Huybrechts M, Vangronsveld J, Gielen M, Keunen E, Cuypers A. Reciprocal Interactions between Cadmium-Induced Cell Wall Responses and Oxidative Stress in Plants. *Front Plant Sci*. 2017;8. doi:10.3389/fpls.2017.01867

6. Altschul SF, Madden TL, Schäffer AA, Zhang J, Zhang Z, Miller W, Lipman DJ. Gapped BLAST and PSI-BLAST: a new generation of protein database search programs. *Nucleic Acids Res*. 1997;25(17):3389-3402. doi:10.1093/nar/25.17.3389
